# Plasma nervonic acid levels were negatively associated with attention levels in community-living older adults in New Zealand

**DOI:** 10.1007/s11306-022-01908-5

**Published:** 2022-07-16

**Authors:** Jamie V de Seymour, Kathryn L Beck, Cathryn A Conlon, Pamela R von Hurst, Karen D Mumme, Crystal F Haskell-Ramsay, Mary Beatrix Jones

**Affiliations:** 1 College of Health, Massey University Auckland, Private Bag 102904, North Shore, 0745 Auckland, New Zealand; 2 Department of Psychology, Northumbria University, NE1 8ST Newcastle, UK; 3 Department of Statistics, University of Auckland, 1010 Auckland, New Zealand

**Keywords:** Metabolomics, Mass spectrometry, Cognition, Healthy aging, Metabolite

## Abstract

The global population is aging. Preserving function and independence of our aging population is paramount. A key component to maintaining independence is the preservation of cognitive function. Metabolomics can be used to identify biomarkers of cognition before noticeable deterioration. Our study investigated the plasma metabolome of 332 community-living New Zealanders between 65 and 74 years of age, using gas chromatography-mass spectrometry. Six cognitive domains were assessed. Of the 123 metabolites identified using an in-house mass spectral libraries of standards, nervonic acid had a significant, inverse association with the attention domain (P-value = 1.52E^− 4^; FDR = 0.019), after adjusting for covariates (apolipoprotein E -ε4 genotype, sex, body fat percentage (standardised by sex), age, education, deprivation index, physical activity, metabolic syndrome, polypharmacy, smoking status, and alcohol intake) and multiple testing. Attention is defined as the ability to concentrate on selected aspects of the environment while ignoring other stimuli. This is the first study to identify nervonic acid as a potential biomarker of attention in older adults. Future research should confirm this association in a longitudinal study.

**Supplementary Information:**

The online version contains supplementary material available at 10.1007/s11306-022-01908-5.

## Introduction

The global population is aging. By 2050, an estimated 16% of the worldwide population will be over 65 (compared to 9% in 2019) (United Nations Department of Economic and Social Affairs, 2019). Preserving function and independence of our aging population is paramount for ensuring quality of life and reducing the pressures on society of an aging population. A key component of good health is cognitive function.

The assessment of cognition in older adults typically involves screening tools administered by trained physicians (*Assessing Cognitive Impairment in Older Patients | National Institute on Aging*, 2021). In many instances, these are not routinely performed and only occur after signs/symptoms of cognitive impairment are observed. Ideally, any cognitive decline or evidence of impairment should be identified at the earliest stage.

Assessment tools currently used for early screening can lack sensitivity to detect early cognitive decline, are affected by education, and can be insensitive to right hemisphere dysfunction (Chan et al., 2017; Wu et al., 2013). Biological markers may provide an objective alternative to measuring cognitive function in ‘healthy’ individuals. Identifying individuals at risk of cognitive decline early would allow for interventions to preserve cognitive function and maintain independence.

Metabolomics, the study of low weight compounds in a biological system, has been used widely as a tool for biomarker investigations (Bracewell-Milnes et al., 2017; Gibbons et al., 2017). A biomarker, as defined by the United States Food and Drug Administration, is “a defined characteristic that is measured as an indicator of normal biological processes, pathogenic processes, or responses to an exposure or intervention, including therapeutic interventions” (United States Food and Drug Administration, 2021). Metabolomics is downstream from the other -omics approaches, reflecting the interface between endogenous processes and external exposures (Chin et al., 2013). Several metabolomic studies have identified potential biomarkers of cognition (Jiang et al., 2019; Hosseini et al., 2020). However, they have predominantly focused on the identification of biomarkers for Alzheimer’s Disease and mild cognitive impairment (MCI). In a systematic review by Jiang et al., (2019), a wide range of metabolites were associated with cognitive decline, MCI and dementia though none were consistently identified in the studies. Hosseini et al., (2020) found of 10 studies investigating fatty acids and their associations with MCI, there were few significant findings. However, much like Jiang et al., higher levels of docosahexanoic acid (DHA) were found in control subjects when compared to those with MCI or dementia. Interestingly, Hosseini et al. also found total serum/plasma fatty acids were significantly down-regulated in Alzheimer’s disease.

Our study investigated the plasma metabolome of community-living older adults in New Zealand for biomarkers of cognitive function.

## Materials and methods

### Study design and population

Participants were from the Researching Eating, Activity and Cognitive Health (REACH) cohort (Mumme et al., 2019), all community-living older adults aged 65–74 years, living in Auckland, New Zealand. Participants were excluded from the study if they had a diagnosis of dementia or any of the following conditions which may impair cognitive function: stroke, traumatic head or brain injury, a neurological or psychiatric condition; or if they were taking medication which may influence their cognitive function. Another exclusion criterion was any event in the last 2 years which had a substantial impact on dietary intake and cognitive function, for example, death or illness of a family member. Participants completed demographic, health and lifestyle questionnaires and underwent a Computerised Mental Performance Assessment System (COMPASS-Northumbria University, Newcastle upon Tyne, UK) battery of cognitive tests assessing five domains (Supplementary Table 1). The results of these domains were combined to assess overall global cognitive function. The cognitive suite was temperature, noise and light controlled. Participants provided fasted blood samples and were provided a standard breakfast of cereals, fruits, toast and spreads prior to cognition testing. Informed consent was obtained from participants and ethics approval was received from the Massey University Human Ethics Committee: Southern A, Application 17/69.

### Metabolomic analysis

#### Fatty acid methyl esters (FAMEs) method

The FAMEs extraction and derivatisation method was based on the protocol in LePage & Roy (Lepage & Roy, 1986), using a 50 µL plasma aliquot from each participant. Gas chromatography-mass spectrometry (GC-MS) analysis was performed on an Agilent 7890 A gas chromatograph coupled to a 5975 C mass spectrometer with a split/splitless inlet using instrument analytical parameters published in Kramer et al. (*Official Methods for Determination of trans Fat, Second Edition − 2nd*, 2010). GC-MS parameters are detailed in Supplementary Information 1.

#### Methyl chloroformate (MCF) method

Prior to MCF derivatisation, a 300 µL plasma aliquot from each participant underwent cold methanol extraction using 50 and 80%v/v methanol/water. The MCF derivatisation procedure and analytical parameters used for GC-MS analysis are published in Smart et al. (Smart et al., 2010). GC-MS parameters are detailed in Supplementary Information 1.

#### Data processing

Batch processing of raw data was conducted using the automated mass spectral deconvolution and identification system software, using parameters detailed in Supplementary Information 2.

Identification of metabolites were based on accurate reference ion mass and retention-time information using in-house mass spectral libraries of standards. An in-house R package “MassOmics” was used for metabolite identification and automated peak integration (https://github.com/MASHUOA/MassOmics). Peak responses were normalized by internal standards (nonadecanoic acid in the FAMEs method, d4-Alanine in the MCF method) and contaminants were identified using experimental “blank” samples and corrected for. Median centring was performed to adjust for batch effects, with a multiplicative correction.

### Statistical analysis

Statistical analyses were performed using R software, version 4.0.2. False discovery rates (FDR) were calculated for all metabolomic comparisons using the Benjamini-Hochberg procedure (repeated for each cognitive domain), and FDR < 0.05 were deemed statistically significant. Both unadjusted and adjusted multivariate regression analyses were performed to determine which metabolites had associations with cognitive scores (an excerpt of the R script can be found in Supplementary Information 3). Analyses were adjusted for: Apolipoprotein E -ε4 (*APOE*) genotype, sex, body fat percentage (standardised by sex), age, education, deprivation index, physical activity, metabolic syndrome, polypharmacy, smoking status, and alcohol intake (details and justifications for including each component can be found in Supplementary Information 4).

## Results

### Participant characteristics

Participants were included if they had scores on all COMPASS domains, provided a blood sample, and had sufficient plasma volume for analysis. Fourteen samples were excluded based on visual assessment of the principal component analysis plot of standardized metabolite scores (Supplementary Information 5). Principal component analysis was used to visualise the combination of metabolite features rather than identifying outliers based on measures of individual metabolites as variation in some metabolites can be biologically plausible, whereas a range of abnormal levels is more likely to indicate a technical abnormality that could influence results. A total of 332 participants were included in the final analysis. The mean ± SD age of participants was 69.7 ± 2.6 years; body fat percentage was 31.8 ± 7.5%; 63.3% were female, 92.2% were of New Zealand European ethnicity; and 27.7% carried at least one *ε4* allele on the *APOE* gene.

### Metabolomics findings

A total of 42 fatty acids were identified in the plasma samples following the FAMEs method and 81 metabolites following the MCF method. In the unadjusted models, there were 35 metabolite-cognition associations with a P-value < 0.05 (Supplementary Table 2). In the models adjusted for confounding variables, there were 29 metabolite-cognition associations with a P-value < 0.05 (Supplementary Table 3). After accounting for multiple comparisons, only one metabolite-cognition association remained statistically significant (FDR < 0.05): 15-tetracosenoic acid demonstrated an inverse linear association with attention (P-value = 9.36E^− 5^; FDR = 0.012), which remained significant after adjustment for confounders (P-value = 1.52E^− 4^; FDR = 0.019). Table 1 displays the final model from the multivariate linear regression of 15-tetracosenoic acid with attention.

**Table 1:** Multivariate linear regression of 15-tetracosenoic acid z-scores and attention z-scores, adjusted for confounders

	Β^1^ (95% CI)	P-value
15-tetracosenoic acid	-0.211 (-0.320, -0.103)	< 0.001
Age	-0.037 (-0.08, 0.002)	0.065
Sex Male (reference) Female	-0.011 (-0.251, 0.230)	0.930
Deprivation Index	5.40E^− 6^ (7.00E^− 5^, 8.09E^− 5^)	0.888
Apolipoprotein E -ε4 (*APOE*) genotype No allele (reference) One or more allele	0.014 (-0.210, 0.239)	0.899
Body fat percentage (standardised by sex)	-0.005 (-0.027, 0.017)	0.661
Education Secondary or less (reference) Post-secondary University	0.136 (-0.141, 0.413) 0.333 (0.055, 0.611)	0.335 0.019*
Physical activity	-2.46E^− 5^ (-6.17E^− 5^, 1.24E^− 5^)	0.191
Metabolic syndrome Without (reference) With	-0.048 (-0.380, 0.284)	0.778
Polypharmacy <5 medications/day (reference) ≥5 medications/day	-0.048 (-0.427, 0.332)	0.805
Smoking status No/used to (reference) Yes	-0.066 (-0.317, 0.184)	0.602
Alcohol intake	2.57E^− 4^ (-5.34E^− 4^, 0.001)	0.523

CI: Confidence Interval

Adjusted R^2^ for the multiple regression = 0.05

^1^ Beta value

## Discussion

This study is the first to analyse the plasma metabolome for biomarkers of cognition in a cohort of community-living older adults from New Zealand. We found plasma levels of 15-tetracosenoic acid (nervonic acid) had a significant inverse association with attention scores.

The COMPASS assessment of attention captures an individual’s simple reaction time, choice reaction time, and rapid visual information processing. Impairment in these areas of cognition could have severe real-life implications such as an increased risk of collisions while driving, or increased risk of falls (Glisky, 2019). Thus, attention is a cognitive domain with considerable importance to everyday functioning and maintaining independence. Identifying early biomarkers of attention, before impairments result in negative consequences, means at-risk individuals can be monitored and efforts to enhance this cognitive domain initiated.

Impaired attention in older adults has previously been associated with the locus coeruleus, a section of the brainstem (Lee et al., 2018), which has been reported as one of the earliest sites of tau pathology and a key brain region involved in the progression of Alzheimer’s disease (Giorgi et al., 2019). Therefore, early signs of decline in attention could potentially be linked to an increased risk of the eventual development of Alzheimer’s disease.

Nervonic acid is a long-chain omega-9 fatty acid (24:1) which has been found in high levels in brain white matter, in peripheral nerve cells, and is one of the most predominant fatty acids in sphingomyelin (Li et al., 2019;^,^Martínez & Mougan 2002). We propose the observed negative relationship between plasma nervonic acid levels and attention scores may reflect age-related myelin degeneration (Bartzokis, 2004; Peters, 2009), resulting in the leaching of nervonic acid into circulation.

Nervonic acid is also found in some dietary sources, including being incorporated into glycerol esters in some fish oils and in Lunaria annua, a plant of the Brassicaceae family (Akoh et al., 2008; Chow 2008). Therefore, plasma levels may be impacted by dietary consumption. Dietary information was collected in the REACH study and no associations between dietary patterns and cognition were found (Mumme et al., 2022). Therefore, dietary intake was not adjusted for in this study. Plasma is an easy to obtain biospecimen, suitable for use as a screening tool. However, the use of plasma in our study limited our ability to determine metabolic mechanisms underpinning the association between attention and nervonic acid levels. Future studies would benefit from investigations that allow comparisons between nervonic acid levels in plasma and cerebrospinal fluid and/or brain tissue to confirm whether increased nervonic acid levels in plasma are directly related to levels in the brain. Another limitation of our study is the cross-sectional study design. However, the utility of metabolomics to identify midlife plasma metabolites which were able to predict cognitive impairment and dementia in later life (average 17.1 year follow-up) has previously been demonstrated (Bressler et al., 2017). Future studies should consider a longitudinal approach to investigate whether plasma nervonic acid levels reflect not just cognition at the time of sampling, but also serve as a biomarker of future cognition.

## Conclusions

In this study, higher plasma levels of nervonic acid were significantly associated with lower attention scores in community-living older adults. This is the first study to identify nervonic acid as a potential biomarker of attention in older adults. Objective biomarkers of cognition could be used for early identification of individuals at risk of cognitive impairment, allowing for early intervention and an increased likelihood for maintaining independence.

## Electronic supplementary material

Below is the link to the electronic supplementary material.

Supplementary Material 1

## Declarations

### Data sharing statement

Data described in the manuscript will not be available to external parties as study participants did not provide informed consent for this to occur.

## References

[CR1] Akoh, C. C., & Min, D. B. (Eds.). (2008). *Food Lipids: Chemistry, Nutrition, and Biotechnology, Third Edition* (3rd ed.). CRC Press. 10.1201/9781420046649

[CR2] *Assessing Cognitive Impairment in Older Patients | National Institute on Aging* Available at: https://www.nia.nih.gov/health/assessing-cognitive-impairment-older-patients (Accessed: 30 November 2021)

[CR3] Bartzokis, G. (2004). ‘Age-related myelin breakdown: A developmental model of cognitive decline and Alzheimer’s disease’. *Neurobiology of Aging* (pp. 5–18). Elsevier Inc. doi: 10.1016/j.neurobiolaging.2003.03.001 10.1016/j.neurobiolaging.2003.03.001 14675724

[CR4] Bracewell-Milnes T (2017). ’Metabolomics as a tool to identify biomarkers to predict and improve outcomes in reproductive medicine: a systematic review’. Human Reproduction Update.

[CR5] Bressler J (2017). ‘Metabolomics and cognition in African American adults in midlife: the atherosclerosis risk in communities study’, *Translational psychiatry*. Nature Publishing Group.

[CR6] Chan E (2017). ’The test accuracy of the Montreal Cognitive Assessment (MoCA) by stroke lateralisation’. Journal of the neurological sciences.

[CR7] Chin, E., et al. (2013). ‘Applications of metabolomics in food science: Food composition and quality, sensory and nutritional attributes’. *Metabolomics in food and nutrition* (pp. 217–230). Woodhead Publishing

[CR8] Chow CK (2008). Fatty acids in foods and their health implication.

[CR9] Gibbons, H., et al. (2017). ‘Metabolomics as a tool in the identification of dietary biomarkers’, *Proceedings of the Nutrition Society*, 76(1), 42–53 10.1017/S002966511600032X 27221515

[CR10] Giorgi, F. S., et al. (2019). ‘The role of Locus Coeruleus in neuroinflammation occurring in Alzheimer’s disease’. *Brain Research Bulletin* (pp. 47–58). Elsevier Inc. doi: 10.1016/j.brainresbull.2019.08.007 10.1016/j.brainresbull.2019.08.007 31419539

[CR11] Glisky, E. L. (2019). ‘Changes in Cognitive Function in Human Aging’. *Brain Aging* (pp. 3–20). CRC Press. doi: 10.1201/9781420005523-1 21204355

[CR12] Hosseini, M., et al. (2020). ‘Blood fatty acids in Alzheimer’s disease and mild cognitive impairment: A meta-analysis and systematic review’. *Ageing Research Reviews* (p. 101043). Elsevier Ireland Ltd. doi: 10.1016/j.arr.2020.101043 10.1016/j.arr.2020.101043 32194194

[CR13] Jiang, Y., et al. (2019). ‘Metabolomics in the Development and Progression of Dementia: A Systematic Review’, *Frontiers in Neuroscience*. Frontiers Media S.A., 13(APR), p. 343. doi: 10.3389/fnins.2019.00343 10.3389/fnins.2019.00343 PMC6474157 31031585

[CR14] Lee TH (2018). ‘Arousal increases neural gain via the locus coeruleus-noradrenaline system in younger adults but not in older adults’. Nature Human Behaviour Nature Publishing Group.

[CR15] Lepage, G., & Roy, C. C. (1986). ‘Direct transesterification of all classes of lipids in a one-step reaction.’, *Journal of lipid research*. American Society for Biochemistry and Molecular Biology, 27(1), pp. 114–20. Available at: http://www.ncbi.nlm.nih.gov/pubmed/3958609 (Accessed: 4 September 2019) 3958609

[CR16] Li, Q., et al. (2019). ‘A mini review of nervonic acid: Source, production, and biological functions’. *Food Chemistry* (p. 125286). Elsevier Ltd. doi: 10.1016/j.foodchem.2019.125286 10.1016/j.foodchem.2019.125286 31382110

[CR17] Martínez M, Mougan I (2002). ‘Fatty Acid Composition of Human Brain Phospholipids During Normal Development’, *Journal of Neurochemistry*. Lippincott Williams and Wilkins.

[CR18] Mumme KD (2019). ‘Study protocol: associations between dietary patterns, cognitive function and metabolic syndrome in older adults – a cross-sectional study’. BMC Public Health BioMed Central.

[CR19] Mumme KD (2022). ’Dietary patterns and cognitive function in older New Zealand adults: the REACH study’. European Journal of Nutrition.

[CR20] *Official Methods for Determination of trans Fat, Second Edition – 2nd* Available at: https://www.routledge.com/Official-Methods-for-Determination-of-trans-Fat-Second-Edition/Mossoba-Kramer/p/book/9781893997721 (Accessed: 25 August 2020)

[CR21] Peters, A. (2009). ‘The effects of normal aging on myelinated nerve fibers in monkey central nervous system’, *Frontiers in Neuroanatomy*. Frontiers, 3(JUL), p. 11. doi: 10.3389/neuro.05.011.2009 10.3389/neuro.05.011.2009 PMC2713738 19636385

[CR22] Smart, K. F., et al. (2010). ‘Analytical platform for metabolome analysis of microbial cells using methyl chloroformate derivatization followed by gas chromatography–mass spectrometry’, *Nature Protocols*. Nature Publishing Group, 5(10), pp. 1709–1729. doi: 10.1038/nprot.2010.108 10.1038/nprot.2010.108 20885382

[CR23] United Nations Department of Economic and Social Affairs (2019). *World Population Prospects 2019: Highlights*, *United Nations Publication*. Available at: https://population.un.org/wpp/Publications/ (Accessed: 4 October 2020)

[CR24] Unites States Food and Drug Administration (FDA) (2021). *Biomarker terminology: Speaking the same language.* Available at: https://www.fda.gov/files/BIOMARKER-TERMINOLOGY--SPEAKING-THE-SAME-LANGUAGE.pdf (Accessed 24 May 2022)

[CR25] Wu Y (2013). ’The effects of educational background on Montreal Cognitive Assessment screening for vascular cognitive impairment, no dementia, caused by ischemic stroke’. Journal of Clinical Neuroscience.

