# Supplementary material for: Plasma nervonic acid levels were negatively associated with attention levels in community-living older adults in New Zealand
Source: Metabolomics. 2022 Jul 16;18(8):54. doi: 10.1007/s11306-022-01908-5 (PMC9288952; doi:10.1007/s11306-022-01908-5)
Supplement: Supplementary file 1 — Supplementary Material 1 [file 11306_2022_1908_MOESM1_ESM.docx]

**Online Supplementary Material**

Supplementary Table 1. The Computerised Mental Performance Assessment System (COMPASS-Northumbria University, Newcastle upon Tyne, UK) battery of assessments

| Cognitive Domain | Definition | Test |
| --- | --- | --- |
| Attention and vigilance | Attention - ability to concentrate on selected aspects of the environment while ignoring other stimuli  Vigilance - ability to maintain attention and alertness over time | Simple reaction time  Choice reaction time  Digit vigilance task |
| Executive function | Co-ordination of cognitive responses – sub-serves planning, initiating, and inhibiting actions, cognitive flexibility, abstract thinking, and rule acquisition | Stroop test |
| Episodic memory | Ability to retain memories that can be consciously recorded e.g., facts, items, events, faces | Immediate and delayed word recall  Delayed word recognition  Delayed picture recognition |
| Working memory | Ability to hold information in mind while carrying out more complex cognitive processes | Corsi blocks |
| Spatial memory | Assesses visuo-spatial memory | Computerised location learning  Computerised location recall |
| Global cognition | A composite score derived from the scores on the other five domains. |  |

**Supplementary Information 1. Gas chromatography-mass spectrometry parameters**

Fatty acid methyl esters (FAMEs) method

The instrument used was an Agilent 7890A gas chromatograph coupled to a 5975C mass spectrometer with a split/splitless inlet. One microlitre of sample was injected using a CTC PAL autosampler into a glass 4 mm ID straight inlet liner packed with deactivated glass wool (Restek Sky®). In splitless mode, the inlet temperature was 250°C, the column flow was set at 1 mL/min, with a column head pressure of 9 psi, giving an average linear velocity of 19 cm/sec. Purge flow was set to 50 mL/min 1 min after injection.

Column selection was based on the recommendations from the Official Methods for the determination of trans fat (American Oil Chemists Society)^1^. The column was a fused silica Rtx-2330 100 m long, 0.25 mm internal diameter, 0.2 µm highly polar stationary phase (90% biscyanopropyl 10% cyanopropylphenyl polysiloxane, Shimadzu). Carrier gas was instrument grade helium (99.99%, BOC). The gas chromatograph oven temperature programming started isothermally at 45°C for 2 minutes, increased 10°C/min to 215°C, held 35 min; increased 40°C/min to 250°C and held 10 minutes.  The transfer line to the mass spectrometric detector (MSD) was maintained at 250°C, the MSD source at 230°C and the MSD quadropole at 150°C. The detector was turned on 14.5 minutes into the run. The detector was run in positive-ion, electron-impact ionisation mode, at 70 eV electron energy, with electron multiplier set with no additional voltage relative to the autotune value. Data was acquired at 1463 amu/s in scan mode from 41 to 420 atomic mass units, with a detection threshold of 100 ion counts.

1 Mossoba, M. M. & Kramer, J. K. G. *Official methods for the determination of trans fat*. 2nd edn, (AOCS Press, 2009).

Methyl chloroformate (MCF) method

The instrument used was an Agilent 7890A gas chromatograph coupled to a 5975C inert mass spectrometer with a split/splitless inlet. One microliter of sample was injected using a CTC PAL autosampler into a glass split/splitless 4 mm ID straight inlet liner packed with deactivated glass wool (Supelco). The inlet was set to 290°C, pulsed splitless at 180 kPa for 1 min, pressure 56.76 kPa, column flow 1.0 mL/min, giving a calculated average initial linear velocity of 35 cm/sec. Purge flow was set to 25 mL/min 1.1 minute after injection.

The column was a fused silica ZB-1701 30m long, 0.25mm internal diameter, 0.15 µm stationary phase (86% dimethylpolysiloxane, 14% cyanopropylphenyl, Phenomenex). Carrier gas was ultra-high purity grade helium (99.9999%, BOC). Gas chromatograph oven temperature programming started isothermally at 45°C for 2 minutes, increased 9°C/min to 180°C, held 5 minutes; increased 40°C/min to 220°C, held 5 minutes; increased 40°C/min to 240°C, held 11.5 minutes; increased 40°C/min to 280°C, and held 2 minutes. The transfer line to the MSD was maintained at 250°C, the source at 230°C and quadropole at 150°C. The detector was turned on 5.5 minutes into the run. The detector was run in positive-ion, electron-impact ionisation mode, at 70 eV electron energy, with electron multiplier set with no additional voltage relative to the autotune value. N-hexane blanks were run for every 10-12 samples to monitor instrument carryover. Identification of compounds was carried out using mass spectra acquired in scan mode from 38 to 550 amu, with detection threshold of 100 ion counts.

**Supplementary Information 2. Metabolomics data processing parameters**

Methyl chloroformate (MCF) method

Batch processing of raw data from GC-MS analysis was conducted using the AMDIS software (automated mass spectral deconvolution and identification system).

AMDIS settings:
Minimum match factor = 65
Resolution = Medium
Sensitivity = Medium
Shape requirements = Medium
Adjacent peak subtraction = Two

Identification of metabolites were based on accurate reference ion mass and retention-time information using an in-house MCF mass spectral library of standards. An in-house R package “MassOmics” was used for metabolite identification and automated peak integration. Peak responses were normalized by the intensity of d4-Alanine (internal standard), and blank treatment was applied to identify contaminants introduced during sample preparation and correct for the baseline peak response using experimental “blank” samples. Contaminants identified (defined as >30% missing values) were removed from the final data set, and negative values resulting from blank treatment was replaced with zero.

Fatty acid methyl esters (FAMEs) method

Batch processing of raw data from GC-MS analysis was conducted using the AMDIS software (automated mass spectral deconvolution and identification system).

AMDIS settings:
Minimum match factor = 65
Resolution = Medium
Sensitivity = Medium
Shape requirements = Medium
Adjacent peak subtraction = Two

Identification of metabolites were based on accurate reference ion mass and retention-time information using an in-house FAMEs mass spectral library of standards. An in-house R package “MassOmics” was used for metabolite identification and automated peak integration. Peak responses were normalized by the intensity of Nonadecanoic acid (C19 - internal standard), and blank treatment was applied to identify contaminants introduced during sample preparation and correct for the baseline peak response using experimental “blank” samples. Contaminants identified (defined as >30% missing values) were removed from the final data set, and negative values resulting from blank treatment was replaced with zero.

**Supplementary Information 3. An excerpt of the R script for regression analyses**

library(tidyverse)

data.df <- as.data.frame(read_rds(path="15 Metabolites and CogF/data/15 Metabolites data.rds"))

# 5.0 regression - Model 5

##Cognitive function and metabolites with ApoE, sex, body fat (adjusted for sex [residual method]), age, education, deprivation, physical activity, metabolic syndrome, polypharmacy, smoking status, alcohol intake as confounders

## z Global function

cogf = "zglobal"

metab.name <- 0

fit.m <- 0 # set up for model number output

fit.o <- 0 # set up for cognitive outcome

var <- " " # set up for variable confounder

fit.b <- 0 # set up for beta value output

fit.CIl <- 0 # set up for beta 2.5% CI

fit.CIh <- 0 # set up for beta 97.5% CI

fit.p <- 0 # set up for p value output

fit.adjR <- 0 #set up for model adjusted R value output

fit.apoe <- 0 #set up for variable 1

fit.apoe.b <- 0 # set up for beta value for apoe

fit.apoe.CIl <- 0 # set up for beta 2.5% CI

fit.apoe.CIh <- 0 # set up for beta 97.5% CI

fit.apoe.p <- 0 # set up for p value for apoe

fit.sex <- 0 #set up for variable 1

fit.sex.b <- 0 # set up for beta value for sex

fit.sex.CIl <- 0 # set up for beta 2.5% CI

fit.sex.CIh <- 0 # set up for beta 97.5% CI

fit.sex.p <- 0 # set up for p value for sex

fit.bf <- 0 #set up for variable 1

fit.bf.b <- 0 # set up for beta value for body fat%

fit.bf.CIl <- 0 # set up for beta 2.5% CI

fit.bf.CIh <- 0 # set up for beta 97.5% CI

fit.bf.p <- 0 # set up for p value for body fat%

fit.age <- 0 #set up for variable 1

fit.age.b <- 0 # set up for beta value for age

fit.age.CIl <- 0 # set up for beta 2.5% CI

fit.age.CIh <- 0 # set up for beta 97.5% CI

fit.age.p <- 0 # set up for p value for age

fit.educatio <- 0 #set up for variable 1

fit.educatio.b <- 0 # set up for beta value for education

fit.educatio.CIl <- 0 # set up for beta 2.5% CI

fit.educatio.CIh <- 0 # set up for beta 97.5% CI

fit.educatio.p <- 0 # set up for p value for education

fit.imd <- 0 #set up for variable 1

fit.imd.b <- 0 # set up for beta value for deprivation score

fit.imd.CIl <- 0 # set up for beta 2.5% CI

fit.imd.CIh <- 0 # set up for beta 97.5% CI

fit.imd.p <- 0 # set up for p value for deprivation score

fit.pa <- 0 #set up for variable 1

fit.pa.b <- 0 # set up for beta value for physical activity

fit.pa.CIl <- 0 # set up for beta 2.5% CI

fit.pa.CIh <- 0 # set up for beta 97.5% CI

fit.pa.p <- 0 # set up for p value for physical activity

fit.MetS <- 0 #set up for variable 1

fit.MetS.b <- 0 # set up for beta value for metabolic syndrome

fit.MetS.CIl <- 0 # set up for beta 2.5% CI

fit.MetS.CIh <- 0 # set up for beta 97.5% CI

fit.MetS.p <- 0 # set up for p value for metabolic syndrome

fit.polypharma <- 0 #set up for variable 1

fit.polypharma.b <- 0 # set up for beta value for polypharmacy

fit.polypharma.CIl <- 0 # set up for beta 2.5% CI

fit.polypharma.CIh <- 0 # set up for beta 97.5% CI

fit.polypharma.p <- 0 # set up for p value for polypharmacy

fit.smoke <- 0 #set up for variable 1

fit.smoke.b <- 0 # set up for beta value for smoking status

fit.smoke.CIl <- 0 # set up for beta 2.5% CI

fit.smoke.CIh <- 0 # set up for beta 97.5% CI

fit.smoke.p <- 0 # set up for p value for smoking status

fit.alc <- 0 #set up for variable 1

fit.alc.b <- 0 # set up for beta value for alcohol beverage intake

fit.alc.CIl <- 0 # set up for beta 2.5% CI

fit.alc.CIh <- 0 # set up for beta 97.5% CI

fit.alc.p <- 0 # set up for p value for alcohol beverage intake

fit.edu.s <- 0 #set up for variable 1

fit.edu.s.b <- 0 # set up for beta value for education - postsecondary

fit.edu.s.CIl <- 0 # set up for beta 2.5% CI

fit.edu.s.CIh <- 0 # set up for beta 97.5% CI

fit.edu.s.p <- 0 # set up for p value for alcohol education - postsecondary

fit.edu.u <- 0 #set up for variable 1

fit.edu.u.b <- 0 # set up for beta value for education - university

fit.edu.u.CIl <- 0 # set up for beta 2.5% CI

fit.edu.u.CIh <- 0 # set up for beta 97.5% CI

fit.edu.u.p <- 0 # set up for p value for alcohol education - university

for(i in 2:127){

stdmetab <- as.vector(data.df[i])

mname <- colnames(stdmetab)

metab.name[[i-1]] <- mname

working <- bind_cols(stdmetab, data.df[c("zglobal", "apoe", "sex", "body_fat.resid", "age", "educatio", "imd_rank", "pa_score", "MetS", "polypharma", "smoke", "totalalc.ei.adj")])

print(paste(mname, " and ", cogf, " (with ApoE, sex, body fat (standardised by sex), age, education, deprivation score, physical activity score, metabolic syndrome, polypharmacy, smoking status, alcohol beverage intake - model 5"))

fit <- lm(zglobal~working[,1] + apoe + sex + body_fat.resid + age + imd_rank + pa_score + MetS + polypharma + smoke + totalalc.ei.adj + educatio, data=working) # model 5

fit.sum <- summary(fit)

print (fit.sum)

print(round(confint(fit),4))

fit.m[[i-1]] <- 5 # model 5

fit.o[[i-1]] <- cogf # model 5

fit.b[[i-1]] <- fit.sum$coefficients[2,1] #cognition ~ metabolite : beta

fit.CIl[[i-1]] <- confint(fit)[2,1] #cognition ~ metabolite : 2.5% CI

fit.CIh[[i-1]] <- confint(fit)[2,2] #cognition ~ metabolite : 97.5% CI

fit.p[[i-1]] <- fit.sum$coefficients[2,4] #cognition ~ metabolite : p-value

fit.adjR[[i-1]] <- fit.sum$adj.r.squared #cognition ~ metabolite : adj R^2

fit.apoe[[i-1]] <- "ApoE"

fit.apoe.b[[i-1]] <- fit.sum$coefficients[3,1] #cognition ~ ApoE : beta

fit.apoe.CIl[[i-1]] <- confint(fit)[3,1] #cognition ~ ApoE : 2.5% CI

fit.apoe.CIh[[i-1]] <- confint(fit)[3,2] #cognition ~ ApoE : 97.5% CI

fit.apoe.p[[i-1]] <- fit.sum$coefficients[3,4] #cognition ~ ApoE : p-value

fit.sex[[i-1]] <- "sex"

fit.sex.b[[i-1]] <- fit.sum$coefficients[4,1] #cognition ~ sex : beta

fit.sex.CIl[[i-1]] <- confint(fit)[4,1] #cognition ~ sex : 2.5% CI

fit.sex.CIh[[i-1]] <- confint(fit)[4,2] #cognition ~ sex : 97.5% CI

fit.sex.p[[i-1]] <- fit.sum$coefficients[4,4] #cognition ~ sex : p-value

fit.bf[[i-1]] <- "body fat"

fit.bf.b[[i-1]] <- fit.sum$coefficients[5,1] #cognition ~ bf : beta

fit.bf.CIl[[i-1]] <- confint(fit)[5,1] #cognition ~ bf : 2.5% CI

fit.bf.CIh[[i-1]] <- confint(fit)[5,2] #cognition ~ bf : 97.5% CI

fit.bf.p[[i-1]] <- fit.sum$coefficients[5,4] #cognition ~ bf : p-value

fit.age[[i-1]] <- "age"

fit.age.b[[i-1]] <- fit.sum$coefficients[6,1] #cognition ~ age : beta

fit.age.CIl[[i-1]] <- confint(fit)[6,1] #cognition ~ age : 2.5% CI

fit.age.CIh[[i-1]] <- confint(fit)[6,2] #cognition ~ age : 97.5% CI

fit.age.p[[i-1]] <- fit.sum$coefficients[6,4] #cognition ~ age : p-value

fit.imd[[i-1]] <- "IMD"

fit.imd.b[[i-1]] <- fit.sum$coefficients[7,1] #cognition ~ imd : beta

fit.imd.CIl[[i-1]] <- confint(fit)[7,1] #cognition ~ imd : 2.5% CI

fit.imd.CIh[[i-1]] <- confint(fit)[7,2] #cognition ~ imd : 97.5% CI

fit.imd.p[[i-1]] <- fit.sum$coefficients[7,4] #cognition ~ imd : p-value

fit.pa[[i-1]] <- "physical activity"

fit.pa.b[[i-1]] <- fit.sum$coefficients[8,1] #cognition ~ pa_score : beta

fit.pa.CIl[[i-1]] <- confint(fit)[8,1] #cognition ~ pa_score : 2.5% CI

fit.pa.CIh[[i-1]] <- confint(fit)[8,2] #cognition ~ pa_score : 97.5% CI

fit.pa.p[[i-1]] <- fit.sum$coefficients[8,4] #cognition ~ pa_score : p-value

fit.MetS[[i-1]] <- "MetS"

fit.MetS.b[[i-1]] <- fit.sum$coefficients[9,1] #cognition ~ MetS : beta

fit.MetS.CIl[[i-1]] <- confint(fit)[9,1] #cognition ~ MetS : 2.5% CI

fit.MetS.CIh[[i-1]] <- confint(fit)[9,2] #cognition ~ MetS : 97.5% CI

fit.MetS.p[[i-1]] <- fit.sum$coefficients[9,4] #cognition ~ MetS : p-value

fit.polypharma[[i-1]] <- "polypharma"

fit.polypharma.b[[i-1]] <- fit.sum$coefficients[10,1] #cognition ~ polypharma : beta

fit.polypharma.CIl[[i-1]] <- confint(fit)[10,1] #cognition ~ polypharma : 2.5% CI

fit.polypharma.CIh[[i-1]] <- confint(fit)[10,2] #cognition ~ polypharma : 97.5% CI

fit.polypharma.p[[i-1]] <- fit.sum$coefficients[10,4] #cognition ~ polypharma : p-value

fit.smoke[[i-1]] <- "smoke"

fit.smoke.b[[i-1]] <- fit.sum$coefficients[11,1] #cognition ~ smoke : beta

fit.smoke.CIl[[i-1]] <- confint(fit)[11,1] #cognition ~ smoke : 2.5% CI

fit.smoke.CIh[[i-1]] <- confint(fit)[11,2] #cognition ~ smoke : 97.5% CI

fit.smoke.p[[i-1]] <- fit.sum$coefficients[11,4] #cognition ~ smoke : p-value

fit.alc[[i-1]] <- "alcohol"

fit.alc.b[[i-1]] <- fit.sum$coefficients[12,1] #cognition ~ alc : beta

fit.alc.CIl[[i-1]] <- confint(fit)[12,1] #cognition ~ alc : 2.5% CI

fit.alc.CIh[[i-1]] <- confint(fit)[12,2] #cognition ~ alc : 97.5% CI

fit.alc.p[[i-1]] <- fit.sum$coefficients[12,4] #cognition ~ alc : p-value

fit.edu.s[[i-1]] <- "education post secondary"

fit.edu.s.b[[i-1]] <- fit.sum$coefficients[13,1] #cognition ~ edu.s : beta

fit.edu.s.CIl[[i-1]] <- confint(fit)[13,1] #cognition ~ edu.s : 2.5% CI

fit.edu.s.CIh[[i-1]] <- confint(fit)[13,2] #cognition ~ edu.s : 97.5% CI

fit.edu.s.p[[i-1]] <- fit.sum$coefficients[13,4] #cognition ~ edu.s : p-value

fit.edu.u[[i-1]] <- "education university"

fit.edu.u.b[[i-1]] <- fit.sum$coefficients[14,1] #cognition ~ edu.u : beta

fit.edu.u.CIl[[i-1]] <- confint(fit)[14,1] #cognition ~ edu.u : 2.5% CI

fit.edu.u.CIh[[i-1]] <- confint(fit)[14,2] #cognition ~ edu.u : 97.5% CI

fit.edu.u.p[[i-1]] <- fit.sum$coefficients[14,4] #cognition ~ edu.u : p-value

print("- - - - - - - - - - - - - - - - - - - - - - - - - - - - - - - - - - - - - - - - - - -")

}

metabolite.cogf <- bind_cols(metab.name, fit.o, var, fit.m,

fit.b, fit.CIl, fit.CIh, fit.p, fit.adjR)

colnames(metabolite.cogf) <- c("Metabolite", "CogF", "Variable", "model #",

"beta", "2.5% CI", "97.5% CI", "p-value", "model adj R")

apoe.cogf <- bind_cols(metab.name, fit.o, fit.apoe, fit.m,

fit.apoe.b, fit.apoe.CIl, fit.apoe.CIh, fit.apoe.p, fit.adjR)

colnames(apoe.cogf) <- c("Metabolite", "CogF","Variable", "model #",

"beta", "2.5% CI", "97.5% CI", "p-value", "model adj R")

sex.cogf <- bind_cols(metab.name, fit.o, fit.sex, fit.m,

fit.sex.b, fit.sex.CIl, fit.sex.CIh, fit.sex.p, fit.adjR)

colnames(sex.cogf) <- c("Metabolite", "CogF","Variable", "model #",

"beta", "2.5% CI", "97.5% CI", "p-value", "model adj R")

bf.cogf <- bind_cols(metab.name, fit.o, fit.bf, fit.m,

fit.bf.b, fit.bf.CIl, fit.bf.CIh, fit.bf.p, fit.adjR)

colnames(bf.cogf) <- c("Metabolite", "CogF","Variable", "model #",

"beta", "2.5% CI", "97.5% CI", "p-value", "model adj R")

age.cogf <- bind_cols(metab.name, fit.o, fit.age, fit.m,

fit.age.b, fit.age.CIl, fit.age.CIh, fit.age.p, fit.adjR)

colnames(age.cogf) <- c("Metabolite", "CogF","Variable", "model #",

"beta", "2.5% CI", "97.5% CI", "p-value", "model adj R")

imd.cogf <- bind_cols(metab.name, fit.o, fit.imd, fit.m,

fit.imd.b, fit.imd.CIl, fit.imd.CIh, fit.imd.p, fit.adjR)

colnames(imd.cogf) <- c("Metabolite", "CogF","Variable", "model #",

"beta", "2.5% CI", "97.5% CI", "p-value", "model adj R")

pa.cogf <- bind_cols(metab.name, fit.o, fit.pa, fit.m,

fit.pa.b, fit.pa.CIl, fit.pa.CIh, fit.pa.p, fit.adjR)

colnames(pa.cogf) <- c("Metabolite", "CogF","Variable", "model #",

"beta", "2.5% CI", "97.5% CI", "p-value", "model adj R")

MetS.cogf <- bind_cols(metab.name, fit.o, fit.MetS, fit.m,

fit.MetS.b, fit.MetS.CIl, fit.MetS.CIh, fit.MetS.p, fit.adjR)

colnames(MetS.cogf) <- c("Metabolite", "CogF","Variable", "model #",

"beta", "2.5% CI", "97.5% CI", "p-value", "model adj R")

polypharma.cogf <- bind_cols(metab.name, fit.o, fit.polypharma, fit.m,

fit.polypharma.b, fit.polypharma.CIl, fit.polypharma.CIh, fit.polypharma.p, fit.adjR)

colnames(polypharma.cogf) <- c("Metabolite", "CogF","Variable", "model #",

"beta", "2.5% CI", "97.5% CI", "p-value", "model adj R")

smoke.cogf <- bind_cols(metab.name, fit.o, fit.smoke, fit.m,

fit.smoke.b, fit.smoke.CIl, fit.smoke.CIh, fit.smoke.p, fit.adjR)

colnames(smoke.cogf) <- c("Metabolite", "CogF","Variable", "model #",

"beta", "2.5% CI", "97.5% CI", "p-value", "model adj R")

alc.cogf <- bind_cols(metab.name, fit.o, fit.alc, fit.m,

fit.alc.b, fit.alc.CIl, fit.alc.CIh, fit.alc.p, fit.adjR)

colnames(alc.cogf) <- c("Metabolite", "CogF","Variable", "model #",

"beta", "2.5% CI", "97.5% CI", "p-value", "model adj R")

edu.s.cogf <- bind_cols(metab.name, fit.o, fit.edu.s, fit.m,

fit.edu.s.b, fit.edu.s.CIl, fit.edu.s.CIh, fit.edu.s.p, fit.adjR)

colnames(edu.s.cogf) <- c("Metabolite", "CogF","Variable", "model #",

"beta", "2.5% CI", "97.5% CI", "p-value", "model adj R")

edu.u.cogf <- bind_cols(metab.name, fit.o, fit.edu.u, fit.m,

fit.edu.u.b, fit.edu.u.CIl, fit.edu.u.CIh, fit.edu.u.p, fit.adjR)

colnames(edu.u.cogf) <- c("Metabolite", "CogF","Variable", "model #",

"beta", "2.5% CI", "97.5% CI", "p-value", "model adj R")

zglobal.m5 <- bind_rows(metabolite.cogf, apoe.cogf, sex.cogf, bf.cogf, age.cogf, imd.cogf, pa.cogf, MetS.cogf, polypharma.cogf, smoke.cogf, alc.cogf, edu.s.cogf, edu.u.cogf)

write_csv(metabolite.cogf, path="/15 Metabolites and CogF/results/supplementary/15 zglobal_metabolite (m5).csv")

write_csv(sex.cogf, path="/15 Metabolites and CogF/results/supplementary/15 zglobal_sex (m5).csv")

write_csv(apoe.cogf, path="/15 Metabolites and CogF/results/supplementary/15 zglobal_apoe (m5).csv")

write_csv(bf.cogf, path="/15 Metabolites and CogF/results/supplementary/15 zglobal_bf (m5).csv")

write_csv(age.cogf, path="/15 Metabolites and CogF/results/supplementary/15 zglobal_age (m5).csv")

write_csv(imd.cogf, path="/15 Metabolites and CogF/results/supplementary/15 zglobal_imd (m5).csv")

write_csv(pa.cogf, path="/15 Metabolites and CogF/results/supplementary/15 zglobal_pa (m5).csv")

write_csv(MetS.cogf, path="/15 Metabolites and CogF/results/supplementary/15 zglobal_MetS (m5).csv")

write_csv(polypharma.cogf, path="/15 Metabolites and CogF/results/supplementary/15 zglobal_polypharma (m5).csv")

write_csv(smoke.cogf, path="/15 Metabolites and CogF/results/supplementary/15 zglobal_smoke (m5).csv")

write_csv(alc.cogf, path="/15 Metabolites and CogF/results/supplementary/15 zglobal_alc (m5).csv")

write_csv(edu.s.cogf, path="/15 Metabolites and CogF/results/supplementary/15 zglobal_edu.s (m5).csv")

write_csv(edu.u.cogf, path="/15 Metabolites and CogF/results/supplementary/15 zglobal_edu.u (m5).csv")

write_csv(zglobal.m5, path="15 metabolites and CogF/results/supplementary/15 zglobal (m5).csv")

**Supplementary Information 4. Confounders**

*APOE* genotyping was performed at Grafton Clinical Genomics, Auckland using MassArray and participants were categorised into two categories (with or without at least one ε4 allele on the *APOE* gene). *APOE* genotyping was included as a confounding variable because the *APOE* ε4 variant significantly increases the risk for developing Alzheimer's disease and is also associated with age-related cognitive decline during normal ageing^5^. Metabolic syndrome was classified using the criteria recommended by the American Heart Association/National Health, Lung and Blood Institute Scientific Statement ^1^; polypharmacy (use of ≥5 medications)^2^, sex, age and smoking status were self-reported; body fat was measured using dual-energy X-ray absorptiometry and this was standardised by sex; education was categorised into either a) secondary education or less, b) post-secondary education, or c) university-level education; deprivation index was determined from the residential address using the New Zealand Index of Multiple Deprivation (IMD) ^3^. Deprivation index was included as a confounding variable as social deprivation is related to cognitive function and decline^6^. Physical activity was assessed using the short-form International Physical Activity Questionnaire^4^ and categorised as low, moderate, or high activity; and alcohol consumption (g/day) was derived from the 109-item food frequency questionnaire and adjusted for energy intake using the residual method.

1. Grundy SM, Cleeman JI, Daniels SR, et al. Diagnosis and management of the metabolic syndrome: An American Heart Association/National Heart, Lung, and Blood Institute scientific statement. *Circulation*. 2005;112(17):2735-2752. doi:10.1161/CIRCULATIONAHA.105.169404

2. Masnoon N, Shakib S, Kalisch-Ellett L, Caughey GE. What is polypharmacy? A systematic review of definitions. *BMC Geriatr* 2017; 17:1-10. https://doi.org/10.1186/s12877-017-0621-2

3. Exeter DJ, Zhao J, Crengle S, Lee A, Browne M. The New Zealand Indices of Multiple Deprivation (IMD): A new suite of indicators for social and health research in Aotearoa, New Zealand. *PLoS One*. 2017;12(8). doi:10.1371/journal.pone.0181260

4. Craig CL, Marshall AL, Sjöström M, et al. International Physical Activity Questionnaire: 12-Country Reliability and Validity. *Med Sci Sport Exerc*. 2003;35(8):1381-1395. doi:10.1249/01.MSS.0000078924.61453.FB

5. Liu, C. C., Kanekiyo, T., Xu, H., Bu, G. Apolipoprotein E and Alzheimer disease: risk, mechanisms and therapy. *Nature Reviews Neurology*. 2013;9(2): 106-118.

6. Hofbauer, L. M., & Rodriguez, F. S. Validation of a social deprivation index and association with cognitive function and decline in older adults. *International Psychogeriatrics*. 2021;33 (12): 1-12.

**Supplementary Information 5. A principal component analysis of all metabolites was performed to visually inspect for outliers.**

After removing outliers, the PCA was reconstructed until no clear outliers remained (all samples were within the boundary of +/- 10).


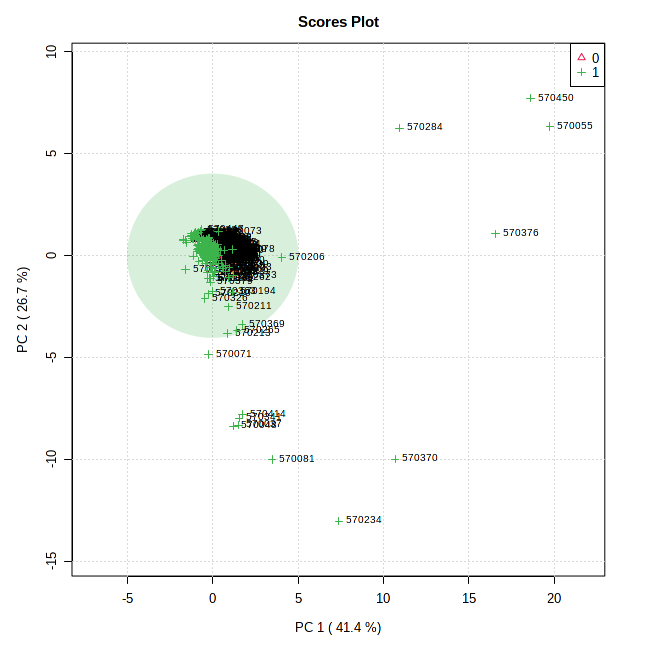

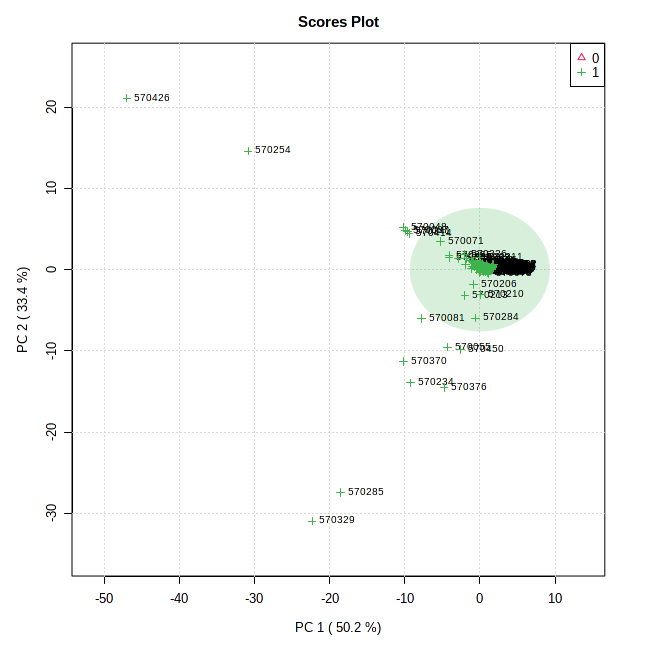

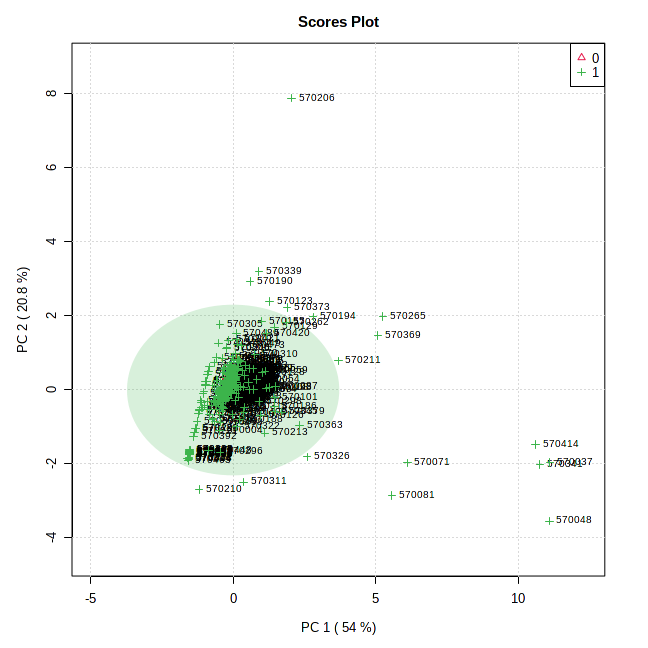


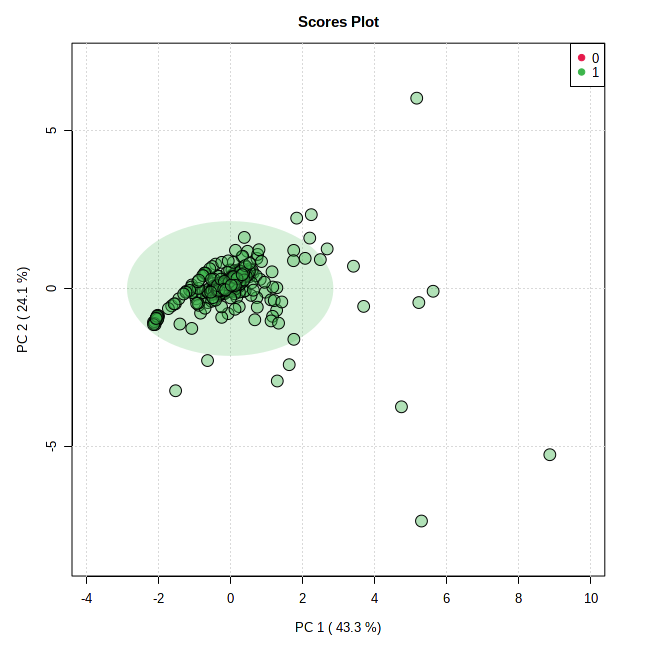


**Supplementary Table 2. The resulting P-values and false discovery rates (FDR) from the unadjusted linear regression between metabolites and each of the cognitive domains**

|  | Global | | Attention and vigilance | | Episodic memory | | Executive function | | Working memory | | Spatial memory | |
| --- | --- | --- | --- | --- | --- | --- | --- | --- | --- | --- | --- | --- |
|  | P-value | FDR | P-value | FDR | P-value | FDR | P-value | FDR | P-value | FDR | P-value | FDR |
| 10-pentadecenoic acid | 0.932 | 0.992 | 0.333 | 0.781 | 0.877 | 0.999 | 0.501 | 0.978 | 0.996 | 0.999 | 0.638 | 0.885 |
| 11,14,17-eicosatrienoic acid | 0.960 | 0.992 | 0.970 | 0.996 | 0.890 | 0.999 | 0.704 | 0.978 | 0.393 | 0.969 | 0.728 | 0.885 |
| 13(E)-docosenoic acid | 0.372 | 0.992 | 0.908 | 0.996 | 0.264 | 0.946 | 0.358 | 0.978 | 0.884 | 0.995 | 0.535 | 0.885 |
| 13(Z)-docosenoic acid | 0.974 | 0.992 | 0.588 | 0.871 | 0.749 | 0.957 | 0.616 | 0.978 | 0.751 | 0.969 | 0.758 | 0.893 |
| 13-docosenomide | 0.370 | 0.992 | 0.288 | 0.781 | 0.915 | 0.999 | 0.111 | 0.850 | 0.578 | 0.969 | 0.426 | 0.885 |
| 15-tetracosenoic acid (nervonic acid) | 0.013 | 0.924 | **9.36E-5** | **0.012** | 0.706 | 0.946 | 0.784 | 0.978 | 0.026 | 0.581 | 0.183 | 0.885 |
| 1-aminocyclopropane-1-carboxylic acid | 0.974 | 0.992 | 0.988 | 0.996 | 0.643 | 0.946 | 0.962 | 0.993 | 0.046 | 0.731 | 0.336 | 0.885 |
| 2-aminobutyric acid | 0.975 | 0.992 | 0.946 | 0.996 | 0.997 | 0.999 | 0.507 | 0.978 | 0.737 | 0.969 | 0.497 | 0.885 |
| 2-aminophenylacetic acid | 0.509 | 0.992 | 0.661 | 0.905 | 0.347 | 0.946 | 0.157 | 0.944 | 0.746 | 0.969 | 0.430 | 0.885 |
| 2-hydroxybutyric acid | 0.426 | 0.992 | 0.939 | 0.996 | 0.251 | 0.946 | 0.926 | 0.985 | 0.479 | 0.969 | 0.674 | 0.885 |
| 2-hydroxyglutaramic acid | 0.506 | 0.992 | 0.771 | 0.962 | 0.608 | 0.946 | 0.201 | 0.978 | 0.855 | 0.995 | 0.620 | 0.885 |
| 2-Hydroxyisobutyric acid | 0.431 | 0.992 | 0.485 | 0.820 | 0.355 | 0.946 | 0.662 | 0.978 | 0.395 | 0.969 | 0.575 | 0.885 |
| 2-oxobutyric acid | 0.309 | 0.992 | 0.825 | 0.990 | 0.193 | 0.946 | 0.527 | 0.978 | 0.445 | 0.969 | 0.340 | 0.885 |
| 2-oxovaleric acid | 0.527 | 0.992 | 0.530 | 0.832 | 0.311 | 0.946 | 0.520 | 0.978 | 0.459 | 0.969 | 0.364 | 0.885 |
| 3-aminoisobutyric acid | 0.433 | 0.992 | 0.050 | 0.673 | 0.719 | 0.954 | 0.845 | 0.978 | 0.727 | 0.969 | 0.774 | 0.903 |
| 3-methyl-2-oxopentanoic acid | 0.449 | 0.992 | 0.039 | 0.673 | 0.765 | 0.964 | 0.970 | 0.994 | 0.810 | 0.990 | 0.290 | 0.885 |
| 4,7,10,13,16,19-docosahexaenoic acid | 0.783 | 0.992 | 0.495 | 0.820 | 0.988 | 0.999 | 0.861 | 0.978 | 0.776 | 0.969 | 0.835 | 0.907 |
| 4,7,10,13,16-docosapentaenoic acid | 0.668 | 0.992 | 0.073 | 0.675 | 0.646 | 0.946 | 0.507 | 0.978 | 0.394 | 0.969 | 0.671 | 0.885 |
| 4-aminobutyric acid | 0.040 | 0.992 | 0.064 | 0.675 | 0.221 | 0.946 | 0.022 | 0.850 | 0.833 | 0.990 | 0.181 | 0.885 |
| 4-hydroxyphenylacetic acid | 0.379 | 0.992 | 0.652 | 0.905 | 0.801 | 0.999 | 0.025 | 0.850 | 0.675 | 0.969 | 0.705 | 0.885 |
| 4-methyl-2-oxopentanoic acid | 0.509 | 0.992 | 0.028 | 0.630 | 0.446 | 0.946 | 0.983 | 0.998 | 0.719 | 0.969 | 0.112 | 0.885 |
| 5,8,11,14,17-eicosapentaenoic acid | 0.326 | 0.992 | 0.211 | 0.781 | 0.484 | 0.946 | 0.532 | 0.978 | 0.621 | 0.969 | 0.833 | 0.907 |
| 5,8,11,14-eicosatetraenoic acid | 0.656 | 0.992 | 0.089 | 0.675 | 0.668 | 0.946 | 0.717 | 0.978 | 0.219 | 0.969 | 0.118 | 0.885 |
| 8,11,14-eicosatrienoic acid | 0.784 | 0.992 | 0.870 | 0.996 | 0.265 | 0.946 | 0.395 | 0.978 | 0.257 | 0.969 | 0.281 | 0.885 |
| 9,12,15-octadecatrienoic acid | 0.182 | 0.992 | 0.082 | 0.675 | 0.749 | 0.957 | 0.427 | 0.978 | 0.028 | 0.581 | 0.804 | 0.907 |
| 9,12-octadecadienoic acid | 0.687 | 0.992 | 0.244 | 0.781 | 0.663 | 0.946 | 0.657 | 0.978 | 0.934 | 0.999 | 0.098 | 0.885 |
| 9-hexadecenoic acid | 0.406 | 0.992 | 0.314 | 0.781 | 0.551 | 0.946 | 0.764 | 0.978 | 0.518 | 0.969 | 0.858 | 0.919 |
| 9-tetradecenoic acid | 0.718 | 0.992 | 0.470 | 0.820 | 0.643 | 0.946 | 0.541 | 0.978 | 0.790 | 0.975 | 0.542 | 0.885 |
| Alanine | 0.605 | 0.992 | 0.694 | 0.916 | 0.554 | 0.946 | 0.740 | 0.978 | 0.652 | 0.969 | 0.679 | 0.885 |
| Asparagine | 0.699 | 0.992 | 0.173 | 0.781 | 0.882 | 0.999 | 0.901 | 0.978 | 0.068 | 0.889 | 0.585 | 0.885 |
| Aspartic acid | 0.585 | 0.992 | 0.798 | 0.981 | 0.303 | 0.946 | 0.413 | 0.978 | 0.470 | 0.969 | 0.615 | 0.885 |
| Azelaic acid | 0.718 | 0.992 | 0.726 | 0.916 | 0.989 | 0.999 | 0.254 | 0.978 | 0.350 | 0.969 | 0.443 | 0.885 |
| beta-alanine | 0.243 | 0.992 | 0.981 | 0.996 | 0.073 | 0.859 | 0.064 | 0.850 | 0.175 | 0.969 | 0.514 | 0.885 |
| Butylated hydroxytoluene | 0.111 | 0.992 | 0.095 | 0.675 | 0.444 | 0.946 | 0.052 | 0.850 | 0.693 | 0.969 | 0.569 | 0.885 |
| Caffeine | 0.585 | 0.992 | 0.462 | 0.820 | 0.462 | 0.946 | 0.071 | 0.850 | 0.439 | 0.969 | 0.835 | 0.907 |
| Cholesterol | 0.839 | 0.992 | 0.208 | 0.781 | 0.449 | 0.946 | 0.906 | 0.978 | 0.017 | 0.581 | 0.249 | 0.885 |
| cis-10-heptadecenoic acid | 0.098 | 0.992 | 0.445 | 0.820 | 0.032 | 0.859 | 0.789 | 0.978 | 0.704 | 0.969 | 0.197 | 0.885 |
| cis-11,14-eicosadienoic acid | 0.181 | 0.992 | 0.243 | 0.781 | 0.187 | 0.946 | 0.858 | 0.978 | 0.991 | 0.999 | 0.285 | 0.885 |
| cis-11-eicosenoic acid | 0.480 | 0.992 | 0.471 | 0.820 | 0.326 | 0.946 | 0.655 | 0.978 | 0.206 | 0.969 | 0.681 | 0.885 |
| cis-11-octadecenoic acid | 0.407 | 0.992 | 0.298 | 0.781 | 0.246 | 0.946 | 0.736 | 0.978 | 0.450 | 0.969 | 0.021 | 0.826 |
| cis-13,16-docasadienoic acid | 0.597 | 0.992 | 0.421 | 0.820 | 0.278 | 0.946 | 0.273 | 0.978 | 0.494 | 0.969 | 0.275 | 0.885 |
| cis-4-hydroxyproline | 0.211 | 0.992 | 0.564 | 0.847 | 0.099 | 0.859 | 0.102 | 0.850 | 0.452 | 0.969 | 0.936 | 0.958 |
| cis-7,10,13,16-docosatetraenoic acid | 0.800 | 0.992 | 0.212 | 0.781 | 0.237 | 0.946 | 0.895 | 0.978 | 0.248 | 0.969 | 0.320 | 0.885 |
| cis-9-octadecenoic acid | 0.847 | 0.992 | 0.234 | 0.781 | 0.705 | 0.946 | 0.908 | 0.978 | 0.475 | 0.969 | 0.314 | 0.885 |
| cis-aconitic acid | 0.864 | 0.992 | 0.631 | 0.898 | 0.976 | 0.999 | 0.586 | 0.978 | 0.761 | 0.969 | 0.085 | 0.885 |
| Citraconic acid | 0.884 | 0.992 | 0.325 | 0.781 | 0.438 | 0.946 | 0.092 | 0.850 | 0.019 | 0.581 | 0.743 | 0.885 |
| Citramalic acid | 0.433 | 0.992 | 0.096 | 0.675 | 0.992 | 0.999 | 0.377 | 0.978 | 0.517 | 0.969 | 0.433 | 0.885 |
| Citric acid | 0.732 | 0.992 | 0.680 | 0.916 | 0.417 | 0.946 | 0.608 | 0.978 | 0.535 | 0.969 | 0.144 | 0.885 |
| Citric acid (secondary peak) | 0.631 | 0.992 | 0.303 | 0.781 | 0.817 | 0.999 | 0.444 | 0.978 | 0.496 | 0.969 | 0.124 | 0.885 |
| Creatinine | 0.841 | 0.992 | 0.450 | 0.820 | 0.572 | 0.946 | 0.560 | 0.978 | 0.668 | 0.969 | 0.192 | 0.885 |
| Cystathionine | 0.881 | 0.992 | 0.157 | 0.781 | 0.697 | 0.946 | 0.833 | 0.978 | 0.109 | 0.969 | 0.449 | 0.885 |
| Cysteine | 0.196 | 0.992 | 0.047 | 0.673 | 0.432 | 0.946 | 0.075 | 0.850 | 0.222 | 0.969 | 0.864 | 0.919 |
| Dibutyl phthalate | 0.463 | 0.992 | 0.486 | 0.820 | 0.632 | 0.946 | 0.121 | 0.850 | 0.291 | 0.969 | 0.199 | 0.885 |
| Dimethyl aminomalonic acid | 0.385 | 0.992 | 0.980 | 0.996 | 0.102 | 0.859 | 0.312 | 0.978 | 0.071 | 0.889 | 0.524 | 0.885 |
| Docosanoic acid | 0.846 | 0.992 | 0.028 | 0.630 | 0.416 | 0.946 | 0.770 | 0.978 | 0.754 | 0.969 | 0.624 | 0.885 |
| Docosapentaenoic acid | 0.438 | 0.992 | 0.370 | 0.820 | 0.064 | 0.859 | 0.702 | 0.978 | 0.732 | 0.969 | 0.467 | 0.885 |
| Dodecanoic acid | 0.678 | 0.992 | 0.115 | 0.738 | 0.995 | 0.999 | 0.565 | 0.978 | 0.893 | 0.995 | 0.488 | 0.885 |
| Eicosanoic acid | 0.934 | 0.992 | 0.399 | 0.820 | 0.148 | 0.946 | 0.094 | 0.850 | 0.885 | 0.995 | 0.632 | 0.885 |
| Ethylenediaminetetraacetic acid | 0.502 | 0.992 | 0.389 | 0.820 | 0.614 | 0.946 | 0.388 | 0.978 | 0.347 | 0.969 | 0.562 | 0.885 |
| Fumaric acid | 0.992 | 0.992 | 0.520 | 0.832 | 0.878 | 0.999 | 0.328 | 0.978 | 0.762 | 0.969 | 0.248 | 0.885 |
| gamma methyl ketoglutaramate | 0.636 | 0.992 | 0.712 | 0.916 | 0.460 | 0.946 | 0.762 | 0.978 | 0.188 | 0.969 | 0.603 | 0.885 |
| gamma-linolenic acid | 0.159 | 0.992 | 0.488 | 0.820 | 0.036 | 0.859 | 0.768 | 0.978 | 0.380 | 0.969 | 0.195 | 0.885 |
| Glutamic acid | 0.935 | 0.992 | 0.977 | 0.996 | 0.570 | 0.946 | 0.333 | 0.978 | 0.948 | 0.999 | 0.247 | 0.885 |
| Glutamine | 0.024 | 0.924 | 0.028 | 0.630 | 0.095 | 0.859 | 0.079 | 0.850 | 0.768 | 0.969 | 0.198 | 0.885 |
| Glutaric acid | 0.338 | 0.992 | 0.939 | 0.996 | 0.142 | 0.946 | 0.952 | 0.991 | 0.137 | 0.969 | 0.635 | 0.885 |
| Glutathione | 0.533 | 0.992 | 0.255 | 0.781 | 0.815 | 0.999 | 0.456 | 0.978 | 0.892 | 0.995 | 0.404 | 0.885 |
| Glycine | 0.379 | 0.992 | 0.635 | 0.898 | 0.042 | 0.859 | 0.439 | 0.978 | 0.098 | 0.969 | 0.466 | 0.885 |
| Glyoxylic acid | 0.846 | 0.992 | 0.237 | 0.781 | 0.218 | 0.946 | 0.725 | 0.978 | 0.508 | 0.969 | 0.619 | 0.885 |
| Heneicosanoic acid | 0.616 | 0.992 | 0.030 | 0.630 | 0.048 | 0.859 | 0.686 | 0.978 | 0.619 | 0.969 | 0.646 | 0.885 |
| Heptadecanoic acid | 0.807 | 0.992 | 0.356 | 0.815 | 0.728 | 0.955 | 0.295 | 0.978 | 0.777 | 0.969 | 0.355 | 0.885 |
| Hexadecanoic acid | 0.253 | 0.992 | 0.083 | 0.675 | 0.638 | 0.946 | 0.547 | 0.978 | 0.881 | 0.995 | 0.697 | 0.885 |
| Hippuric acid | 0.981 | 0.992 | 0.802 | 0.981 | 0.685 | 0.946 | 0.698 | 0.978 | 0.445 | 0.969 | 0.518 | 0.885 |
| Histidine | 0.882 | 0.992 | 0.703 | 0.916 | 0.679 | 0.946 | 0.527 | 0.978 | 0.033 | 0.598 | 0.681 | 0.885 |
| Hydroxybenzoic acid | 0.247 | 0.992 | 0.152 | 0.781 | 0.534 | 0.946 | 0.992 | 0.999 | 0.968 | 0.999 | 0.172 | 0.885 |
| Isocitric acid | 0.406 | 0.992 | 0.562 | 0.847 | 0.291 | 0.946 | 0.895 | 0.978 | 0.574 | 0.969 | 0.736 | 0.885 |
| Isoleucine | 0.734 | 0.992 | 0.314 | 0.781 | 0.476 | 0.946 | 0.817 | 0.978 | 0.532 | 0.969 | 0.137 | 0.885 |
| Itaconic acid | 0.730 | 0.992 | 0.932 | 0.996 | 0.967 | 0.999 | 0.904 | 0.978 | 0.774 | 0.969 | 0.062 | 0.885 |
| Lactic acid | 0.485 | 0.992 | 0.479 | 0.820 | 0.449 | 0.946 | 0.711 | 0.978 | 0.582 | 0.969 | 0.722 | 0.885 |
| Leucine | 0.515 | 0.992 | 0.295 | 0.781 | 0.398 | 0.946 | 0.646 | 0.978 | 0.385 | 0.969 | 0.012 | 0.826 |
| Levulinic acid | 0.275 | 0.992 | 0.460 | 0.820 | 0.415 | 0.946 | 0.055 | 0.850 | 0.490 | 0.969 | 0.302 | 0.885 |
| Lysine | 0.579 | 0.992 | 0.982 | 0.996 | 0.165 | 0.946 | 0.258 | 0.978 | 0.619 | 0.969 | 0.588 | 0.885 |
| Malic acid (peak 1) | 0.713 | 0.992 | 0.961 | 0.996 | 0.447 | 0.946 | 0.859 | 0.978 | 0.342 | 0.969 | 0.140 | 0.885 |
| Malic acid (peak 2) | 0.747 | 0.992 | 0.973 | 0.996 | 0.508 | 0.946 | 0.891 | 0.978 | 0.293 | 0.969 | 0.194 | 0.885 |
| Malonic acid | 0.639 | 0.992 | 0.702 | 0.916 | 0.455 | 0.946 | 0.999 | 0.999 | 0.829 | 0.990 | 0.219 | 0.885 |
| Methionine | 0.681 | 0.992 | 0.053 | 0.673 | 0.182 | 0.946 | 0.061 | 0.850 | 0.720 | 0.969 | 0.585 | 0.885 |
| Methylthioacetic acid | 0.110 | 0.992 | 0.199 | 0.781 | 0.410 | 0.946 | 0.287 | 0.978 | 0.629 | 0.969 | 0.039 | 0.826 |
| Myristic acid | 0.380 | 0.992 | 0.536 | 0.832 | 0.340 | 0.946 | 0.259 | 0.978 | 0.474 | 0.969 | 0.246 | 0.885 |
| Myristoleic acid | 0.944 | 0.992 | 0.997 | 0.997 | 0.836 | 0.999 | 0.751 | 0.978 | 0.539 | 0.969 | 0.613 | 0.885 |
| Nicotinamide | 0.761 | 0.992 | 0.598 | 0.876 | 0.849 | 0.999 | 0.736 | 0.978 | 0.007 | 0.581 | 0.885 | 0.921 |
| Nicotinamide adenine dinucleotide phosphate | 0.686 | 0.992 | 0.385 | 0.820 | 0.991 | 0.999 | 0.595 | 0.978 | 0.608 | 0.969 | 0.526 | 0.885 |
| Nicotinic acid | 0.277 | 0.992 | 0.273 | 0.781 | 0.327 | 0.946 | 0.703 | 0.978 | 0.967 | 0.999 | 0.715 | 0.885 |
| Norvaline | 0.403 | 0.992 | 0.615 | 0.891 | 0.517 | 0.946 | 0.901 | 0.978 | 0.372 | 0.969 | 0.515 | 0.885 |
| Octanoic acid | 0.965 | 0.992 | 0.854 | 0.996 | 0.541 | 0.946 | 0.291 | 0.978 | 0.749 | 0.969 | 0.682 | 0.885 |
| Ornithine | 0.029 | 0.924 | 0.240 | 0.781 | 0.087 | 0.859 | 0.493 | 0.978 | 0.126 | 0.969 | 0.036 | 0.826 |
| Oxalic acid | 0.934 | 0.992 | 0.120 | 0.738 | 0.163 | 0.946 | 0.137 | 0.907 | 0.613 | 0.969 | 0.745 | 0.885 |
| para-toluic acid | 0.705 | 0.992 | 0.333 | 0.781 | 0.221 | 0.946 | 0.678 | 0.978 | 0.305 | 0.969 | 0.831 | 0.907 |
| Pentadecanoic acid | 0.559 | 0.992 | 0.539 | 0.832 | 0.091 | 0.859 | 0.695 | 0.978 | 0.841 | 0.991 | 0.529 | 0.885 |
| Phenethyl acetate | 0.499 | 0.992 | 0.489 | 0.820 | 0.156 | 0.946 | 0.931 | 0.985 | 0.752 | 0.969 | 0.217 | 0.885 |
| Phenylalanine | 0.792 | 0.992 | 0.247 | 0.781 | 0.372 | 0.946 | 0.117 | 0.850 | 0.337 | 0.969 | 0.030 | 0.826 |
| Proline | 0.988 | 0.992 | 0.432 | 0.820 | 0.502 | 0.946 | 0.830 | 0.978 | 0.830 | 0.990 | 0.870 | 0.919 |
| Pyroglutamic acid | 0.375 | 0.992 | 0.079 | 0.675 | 0.638 | 0.946 | 0.870 | 0.978 | 0.226 | 0.969 | 0.389 | 0.885 |
| Pyruvic acid | 0.289 | 0.992 | 0.918 | 0.996 | 0.240 | 0.946 | 0.146 | 0.922 | 0.687 | 0.969 | 0.238 | 0.885 |
| Salicylic acid | 0.019 | 0.924 | 0.225 | 0.781 | 0.083 | 0.859 | 0.108 | 0.850 | 0.015 | 0.581 | 0.357 | 0.885 |
| Serine | 0.504 | 0.992 | 0.960 | 0.996 | 0.313 | 0.946 | 0.404 | 0.978 | 0.546 | 0.969 | 0.508 | 0.885 |
| Stearic acid | 0.799 | 0.992 | 0.717 | 0.916 | 0.933 | 0.999 | 0.576 | 0.978 | 0.643 | 0.969 | 0.358 | 0.885 |
| Suberic acid | 0.739 | 0.992 | 0.954 | 0.996 | 0.579 | 0.946 | 0.942 | 0.989 | 0.918 | 0.999 | 0.800 | 0.907 |
| Succinic acid | 0.645 | 0.992 | 0.220 | 0.781 | 0.954 | 0.999 | 0.838 | 0.978 | 0.920 | 0.999 | 0.811 | 0.907 |
| Tartaric acid | 0.573 | 0.992 | 0.287 | 0.781 | 0.873 | 0.999 | 0.735 | 0.978 | 0.284 | 0.969 | 0.876 | 0.919 |
| Tetracosanoic acid | 0.459 | 0.992 | 0.395 | 0.820 | 0.065 | 0.859 | 0.378 | 0.978 | 0.636 | 0.969 | 0.967 | 0.975 |
| Threonine | 0.389 | 0.992 | 0.299 | 0.781 | 0.467 | 0.946 | 0.837 | 0.978 | 0.190 | 0.969 | 0.116 | 0.885 |
| trans-10-heptadecenoic acid | 0.901 | 0.992 | 0.658 | 0.905 | 0.946 | 0.999 | 0.618 | 0.978 | 0.676 | 0.969 | 0.404 | 0.885 |
| trans-10-nonadecenoic acid | 0.860 | 0.992 | 0.335 | 0.781 | 0.999 | 0.999 | 0.488 | 0.978 | 0.966 | 0.999 | 0.724 | 0.885 |
| trans-11-eicosenoic acid | 0.776 | 0.992 | 0.289 | 0.781 | 0.994 | 0.999 | 0.623 | 0.978 | 0.968 | 0.999 | 0.814 | 0.907 |
| trans-7-nonadecenoic acid | 0.958 | 0.992 | 0.221 | 0.781 | 0.544 | 0.946 | 0.678 | 0.978 | 0.659 | 0.969 | 0.555 | 0.885 |
| trans-9-octadecenoic acid | 0.896 | 0.992 | 0.290 | 0.781 | 0.875 | 0.999 | 0.645 | 0.978 | 0.993 | 0.999 | 0.532 | 0.885 |
| trans-cinnamic acid | 0.915 | 0.992 | 0.295 | 0.781 | 0.675 | 0.946 | 0.707 | 0.978 | 0.999 | 0.999 | 0.238 | 0.885 |
| Tricosanoic acid | 0.520 | 0.992 | 0.835 | 0.993 | 0.275 | 0.946 | 0.695 | 0.978 | 0.200 | 0.969 | 0.427 | 0.885 |
| Tridecane | 0.175 | 0.992 | 0.024 | 0.630 | 0.662 | 0.946 | 0.113 | 0.850 | 0.298 | 0.969 | 0.311 | 0.885 |
| Tryptophan | 0.454 | 0.992 | 0.123 | 0.738 | 0.498 | 0.946 | 0.280 | 0.978 | 0.482 | 0.969 | 0.014 | 0.826 |
| Tyrosine | 0.449 | 0.992 | 0.541 | 0.832 | 0.752 | 0.957 | 0.013 | 0.850 | 0.998 | 0.999 | 0.156 | 0.885 |
| Undecanoic acid | 0.745 | 0.992 | 0.434 | 0.820 | 0.983 | 0.999 | 0.795 | 0.978 | 0.459 | 0.969 | 0.400 | 0.885 |
| Valine | 0.588 | 0.992 | 0.390 | 0.820 | 0.674 | 0.946 | 0.659 | 0.978 | 0.562 | 0.969 | 0.146 | 0.885 |
| Vitamin E | 0.178 | 0.992 | 0.881 | 0.996 | 0.018 | 0.859 | 0.431 | 0.978 | 0.146 | 0.969 | 0.957 | 0.972 |

**Supplementary Table 3.** **The resulting P-values and False Discovery Rates (FDR) from the adjusted multivariate linear regression^1^ between metabolites and each of the cognitive domains**

|  | Global | | Attention and vigilance | | Episodic memory | | Executive function | | Working memory | | Spatial memory | |
| --- | --- | --- | --- | --- | --- | --- | --- | --- | --- | --- | --- | --- |
|  | P-value | FDR | P-value | FDR | P-value | FDR | P-value | FDR | P-value | FDR | P-value | FDR |
| 10-pentadecenoic acid | 0.882 | 0.999 | 0.355 | 0.997 | 0.945 | 0.986 | 0.425 | 0.966 | 0.845 | 0.973 | 0.711 | 0.901 |
| 11,14,17-eicosatrienoic acid | 0.914 | 0.999 | 0.825 | 0.997 | 0.681 | 0.986 | 0.772 | 0.966 | 0.568 | 0.973 | 0.690 | 0.901 |
| 13€-docosenoic acid | 0.515 | 0.999 | 0.857 | 0.997 | 0.577 | 0.986 | 0.452 | 0.966 | 0.916 | 0.973 | 0.568 | 0.901 |
| 13(Z)-docosenoic acid | 0.999 | 0.999 | 0.770 | 0.997 | 0.976 | 0.992 | 0.664 | 0.966 | 0.921 | 0.973 | 0.683 | 0.901 |
| 13-docosenomide | 0.471 | 0.999 | 0.505 | 0.997 | 0.947 | 0.986 | 0.123 | 0.966 | 0.816 | 0.973 | 0.408 | 0.901 |
| 15-tetracosenoic acid (nervonic acid) | 0.001 | 0.099 | **1.52E-04** | **0.019** | 0.079 | 0.821 | 0.458 | 0.966 | 0.048 | 0.516 | 0.151 | 0.901 |
| 1-aminocyclopropane-1-carboxylic acid | 0.721 | 0.999 | 0.814 | 0.997 | 0.771 | 0.986 | 0.749 | 0.966 | 0.012 | 0.369 | 0.433 | 0.901 |
| 2-aminobutyric acid | 0.842 | 0.999 | 0.884 | 0.997 | 0.939 | 0.986 | 0.499 | 0.966 | 0.693 | 0.973 | 0.435 | 0.901 |
| 2-aminophenylacetic acid | 0.993 | 0.999 | 0.713 | 0.997 | 0.857 | 0.986 | 0.283 | 0.966 | 0.873 | 0.973 | 0.630 | 0.901 |
| 2-hydroxybutyric acid | 0.340 | 0.999 | 0.972 | 0.997 | 0.093 | 0.821 | 0.997 | 0.997 | 0.655 | 0.973 | 0.998 | 0.998 |
| 2-hydroxyglutaramic acid | 0.349 | 0.999 | 0.990 | 0.997 | 0.141 | 0.821 | 0.265 | 0.966 | 0.733 | 0.973 | 0.836 | 0.951 |
| 2-Hydroxyisobutyric acid | 0.173 | 0.999 | 0.375 | 0.997 | 0.100 | 0.821 | 0.422 | 0.966 | 0.634 | 0.973 | 0.716 | 0.901 |
| 2-oxobutyric acid | 0.405 | 0.999 | 0.881 | 0.997 | 0.246 | 0.926 | 0.595 | 0.966 | 0.739 | 0.973 | 0.488 | 0.901 |
| 2-oxovaleric acid | 0.425 | 0.999 | 0.509 | 0.997 | 0.169 | 0.821 | 0.540 | 0.966 | 0.561 | 0.973 | 0.319 | 0.901 |
| 3-aminoisobutyric acid | 0.334 | 0.999 | 0.144 | 0.997 | 0.160 | 0.821 | 0.449 | 0.966 | 0.434 | 0.973 | 0.732 | 0.901 |
| 3-methyl-2-oxopentanoic acid | 0.539 | 0.999 | 0.189 | 0.997 | 0.419 | 0.986 | 0.392 | 0.966 | 0.121 | 0.765 | 0.475 | 0.901 |
| 4,7,10,13,16,19-docosahexaenoic acid | 0.868 | 0.999 | 0.608 | 0.997 | 0.986 | 0.993 | 0.872 | 0.966 | 0.846 | 0.973 | 0.886 | 0.955 |
| 4,7,10,13,16-docosapentaenoic acid | 0.181 | 0.999 | 0.035 | 0.997 | 0.636 | 0.986 | 0.953 | 0.969 | 0.172 | 0.918 | 0.732 | 0.901 |
| 4-aminobutyric acid | 0.186 | 0.999 | 0.219 | 0.997 | 0.320 | 0.959 | 0.154 | 0.966 | 0.619 | 0.973 | 0.406 | 0.901 |
| 4-hydroxyphenylacetic acid | 0.903 | 0.999 | 0.847 | 0.997 | 0.612 | 0.986 | 0.202 | 0.966 | 0.923 | 0.973 | 0.755 | 0.914 |
| 4-methyl-2-oxopentanoic acid | 0.515 | 0.999 | 0.138 | 0.997 | 0.750 | 0.986 | 0.514 | 0.966 | 0.195 | 0.943 | 0.171 | 0.901 |
| 5,8,11,14,17-eicosapentaenoic acid | 0.244 | 0.999 | 0.247 | 0.997 | 0.297 | 0.926 | 0.312 | 0.966 | 0.900 | 0.973 | 0.890 | 0.955 |
| 5,8,11,14-eicosatetraenoic acid | 0.362 | 0.999 | 0.153 | 0.997 | 0.632 | 0.986 | 0.536 | 0.966 | 0.263 | 0.973 | 0.169 | 0.901 |
| 8,11,14-eicosatrienoic acid | 0.833 | 0.999 | 0.961 | 0.997 | 0.619 | 0.986 | 0.830 | 0.966 | 0.212 | 0.973 | 0.138 | 0.901 |
| 9,12,15-octadecatrienoic acid | 0.145 | 0.999 | 0.113 | 0.997 | 0.600 | 0.986 | 0.290 | 0.966 | 0.052 | 0.516 | 0.913 | 0.955 |
| 9,12-octadecadienoic acid | 0.884 | 0.999 | 0.335 | 0.997 | 0.785 | 0.986 | 0.663 | 0.966 | 0.594 | 0.973 | 0.103 | 0.901 |
| 9-hexadecenoic acid | 0.515 | 0.999 | 0.602 | 0.997 | 0.524 | 0.986 | 0.701 | 0.966 | 0.691 | 0.973 | 0.737 | 0.901 |
| 9-tetradecenoic acid | 0.824 | 0.999 | 0.828 | 0.997 | 0.602 | 0.986 | 0.546 | 0.966 | 0.608 | 0.973 | 0.492 | 0.901 |
| Alanine | 0.889 | 0.999 | 0.942 | 0.997 | 0.532 | 0.986 | 0.932 | 0.966 | 0.409 | 0.973 | 0.834 | 0.951 |
| Asparagine | 0.939 | 0.999 | 0.546 | 0.997 | 0.771 | 0.986 | 0.551 | 0.966 | 0.023 | 0.423 | 0.645 | 0.901 |
| Aspartic acid | 0.359 | 0.999 | 0.665 | 0.997 | 0.455 | 0.986 | 0.737 | 0.966 | 0.318 | 0.973 | 0.354 | 0.901 |
| Azelaic acid | 0.890 | 0.999 | 0.959 | 0.997 | 0.734 | 0.986 | 0.361 | 0.966 | 0.668 | 0.973 | 0.401 | 0.901 |
| beta-alanine | 0.477 | 0.999 | 0.479 | 0.997 | 0.677 | 0.986 | 0.079 | 0.966 | 0.269 | 0.973 | 0.587 | 0.901 |
| Butylated hydroxytoluene | 0.104 | 0.999 | 0.145 | 0.997 | 0.412 | 0.986 | 0.020 | 0.966 | 0.924 | 0.973 | 0.447 | 0.901 |
| Caffeine | 0.750 | 0.999 | 0.361 | 0.997 | 0.927 | 0.986 | 0.367 | 0.966 | 0.662 | 0.973 | 0.462 | 0.901 |
| Cholesterol | 0.735 | 0.999 | 0.195 | 0.997 | 0.935 | 0.986 | 0.320 | 0.966 | 0.006 | 0.268 | 0.370 | 0.901 |
| cis-10-heptadecenoic acid | 0.076 | 0.999 | 0.274 | 0.997 | 0.051 | 0.821 | 0.711 | 0.966 | 0.953 | 0.973 | 0.256 | 0.901 |
| cis-11,14-eicosadienoic acid | 0.069 | 0.999 | 0.141 | 0.997 | 0.097 | 0.821 | 0.857 | 0.966 | 0.831 | 0.973 | 0.227 | 0.901 |
| cis-11-eicosenoic acid | 0.578 | 0.999 | 0.453 | 0.997 | 0.477 | 0.986 | 0.831 | 0.966 | 0.220 | 0.973 | 0.664 | 0.901 |
| cis-11-octadecenoic acid | 0.358 | 0.999 | 0.456 | 0.997 | 0.256 | 0.926 | 0.890 | 0.966 | 0.349 | 0.973 | 0.032 | 0.901 |
| cis-13,16-docasadienoic acid | 0.853 | 0.999 | 0.427 | 0.997 | 0.640 | 0.986 | 0.298 | 0.966 | 0.552 | 0.973 | 0.328 | 0.901 |
| cis-4-hydroxyproline | 0.256 | 0.999 | 0.560 | 0.997 | 0.203 | 0.880 | 0.054 | 0.966 | 0.443 | 0.973 | 0.956 | 0.980 |
| cis-7,10,13,16-docosatetraenoic acid | 0.557 | 0.999 | 0.146 | 0.997 | 0.345 | 0.986 | 0.818 | 0.966 | 0.138 | 0.826 | 0.457 | 0.901 |
| cis-9-octadecenoic acid | 0.999 | 0.999 | 0.349 | 0.997 | 0.588 | 0.986 | 0.943 | 0.966 | 0.918 | 0.973 | 0.511 | 0.901 |
| cis-aconitic acid | 0.953 | 0.999 | 0.718 | 0.997 | 0.600 | 0.986 | 0.592 | 0.966 | 0.706 | 0.973 | 0.093 | 0.901 |
| Citraconic acid | 0.685 | 0.999 | 0.526 | 0.997 | 0.584 | 0.986 | 0.182 | 0.966 | 0.005 | 0.268 | 0.934 | 0.964 |
| Citramalic acid | 0.510 | 0.999 | 0.221 | 0.997 | 0.664 | 0.986 | 0.536 | 0.966 | 0.176 | 0.918 | 0.524 | 0.901 |
| Citric acid | 0.771 | 0.999 | 0.881 | 0.997 | 0.281 | 0.926 | 0.448 | 0.966 | 0.575 | 0.973 | 0.113 | 0.901 |
| Citric acid (secondary peak) | 0.814 | 0.999 | 0.520 | 0.997 | 0.786 | 0.986 | 0.655 | 0.966 | 0.647 | 0.973 | 0.113 | 0.901 |
| Creatinine | 0.820 | 0.999 | 0.872 | 0.997 | 0.381 | 0.986 | 0.276 | 0.966 | 0.360 | 0.973 | 0.273 | 0.901 |
| Cystathionine | 0.978 | 0.999 | 0.171 | 0.997 | 0.975 | 0.992 | 0.808 | 0.966 | 0.055 | 0.516 | 0.396 | 0.901 |
| Cysteine | 0.744 | 0.999 | 0.151 | 0.997 | 0.958 | 0.989 | 0.206 | 0.966 | 0.042 | 0.516 | 0.705 | 0.901 |
| Dibutyl phthalate | 0.919 | 0.999 | 0.949 | 0.997 | 0.547 | 0.986 | 0.406 | 0.966 | 0.745 | 0.973 | 0.227 | 0.901 |
| Dimethyl aminomalonic acid | 0.871 | 0.999 | 0.967 | 0.997 | 0.900 | 0.986 | 0.492 | 0.966 | 0.087 | 0.647 | 0.673 | 0.901 |
| Docosanoic acid | 0.732 | 0.999 | 0.061 | 0.997 | 0.777 | 0.986 | 0.912 | 0.966 | 0.526 | 0.973 | 0.613 | 0.901 |
| Docosapentaenoic acid | 0.394 | 0.999 | 0.410 | 0.997 | 0.087 | 0.821 | 0.533 | 0.966 | 0.965 | 0.973 | 0.454 | 0.901 |
| Dodecanoic acid | 0.384 | 0.999 | 0.083 | 0.997 | 0.615 | 0.986 | 0.840 | 0.966 | 0.752 | 0.973 | 0.508 | 0.901 |
| Eicosanoic acid | 0.445 | 0.999 | 0.409 | 0.997 | 0.771 | 0.986 | 0.012 | 0.966 | 0.957 | 0.973 | 0.571 | 0.901 |
| Ethylenediaminetetraacetic acid | 0.457 | 0.999 | 0.669 | 0.997 | 0.129 | 0.821 | 0.651 | 0.966 | 0.076 | 0.598 | 0.771 | 0.921 |
| Fumaric acid | 0.697 | 0.999 | 0.766 | 0.997 | 0.165 | 0.821 | 0.228 | 0.966 | 0.745 | 0.973 | 0.119 | 0.901 |
| gamma methyl ketoglutaramate | 0.797 | 0.999 | 0.592 | 0.997 | 0.623 | 0.986 | 0.521 | 0.966 | 0.321 | 0.973 | 0.670 | 0.901 |
| gamma-linolenic acid | 0.068 | 0.999 | 0.469 | 0.997 | 0.010 | 0.650 | 0.826 | 0.966 | 0.469 | 0.973 | 0.185 | 0.901 |
| Glutamic acid | 0.782 | 0.999 | 0.902 | 0.997 | 0.850 | 0.986 | 0.176 | 0.966 | 0.636 | 0.973 | 0.319 | 0.901 |
| Glutamine | 0.139 | 0.999 | 0.218 | 0.997 | 0.116 | 0.821 | 0.440 | 0.966 | 0.259 | 0.973 | 0.261 | 0.901 |
| Glutaric acid | 0.447 | 0.999 | 0.887 | 0.997 | 0.366 | 0.986 | 0.859 | 0.966 | 0.072 | 0.598 | 0.508 | 0.901 |
| Glutathione | 0.365 | 0.999 | 0.601 | 0.997 | 0.289 | 0.926 | 0.362 | 0.966 | 0.354 | 0.973 | 0.459 | 0.901 |
| Glycine | 0.918 | 0.999 | 0.712 | 0.997 | 0.612 | 0.986 | 0.503 | 0.966 | 0.154 | 0.885 | 0.507 | 0.901 |
| Glyoxylic acid | 0.752 | 0.999 | 0.533 | 0.997 | 0.454 | 0.986 | 0.825 | 0.966 | 0.182 | 0.918 | 0.685 | 0.901 |
| Heneicosanoic acid | 0.583 | 0.999 | 0.042 | 0.997 | 0.064 | 0.821 | 0.664 | 0.966 | 0.322 | 0.973 | 0.710 | 0.901 |
| Heptadecanoic acid | 0.758 | 0.999 | 0.413 | 0.997 | 0.758 | 0.986 | 0.274 | 0.966 | 0.623 | 0.973 | 0.386 | 0.901 |
| Hexadecanoic acid | 0.264 | 0.999 | 0.122 | 0.997 | 0.738 | 0.986 | 0.236 | 0.966 | 0.872 | 0.973 | 0.549 | 0.901 |
| Hippuric acid | 0.952 | 0.999 | 0.927 | 0.997 | 0.993 | 0.993 | 0.741 | 0.966 | 0.472 | 0.973 | 0.600 | 0.901 |
| Histidine | 0.719 | 0.999 | 0.989 | 0.997 | 0.832 | 0.986 | 0.917 | 0.966 | 0.015 | 0.387 | 0.787 | 0.921 |
| Hydroxybenzoic acid | 0.458 | 0.999 | 0.126 | 0.997 | 0.773 | 0.986 | 0.862 | 0.966 | 0.860 | 0.973 | 0.191 | 0.901 |
| Isocitric acid | 0.196 | 0.999 | 0.513 | 0.997 | 0.044 | 0.821 | 0.712 | 0.966 | 0.796 | 0.973 | 0.466 | 0.901 |
| Isoleucine | 0.933 | 0.999 | 0.905 | 0.997 | 0.645 | 0.986 | 0.313 | 0.966 | 0.452 | 0.973 | 0.204 | 0.901 |
| Itaconic acid | 0.762 | 0.999 | 0.622 | 0.997 | 0.494 | 0.986 | 0.720 | 0.966 | 0.960 | 0.973 | 0.049 | 0.901 |
| Lactic acid | 0.283 | 0.999 | 0.383 | 0.997 | 0.153 | 0.821 | 0.636 | 0.966 | 0.605 | 0.973 | 0.917 | 0.955 |
| Leucine | 0.512 | 0.999 | 0.992 | 0.997 | 0.640 | 0.986 | 0.921 | 0.966 | 0.851 | 0.973 | 0.013 | 0.901 |
| Levulinic acid | 0.492 | 0.999 | 0.783 | 0.997 | 0.395 | 0.986 | 0.158 | 0.966 | 0.301 | 0.973 | 0.560 | 0.901 |
| Lysine | 0.252 | 0.999 | 0.525 | 0.997 | 0.162 | 0.821 | 0.715 | 0.966 | 0.387 | 0.973 | 0.973 | 0.989 |
| Malic acid (peak 1) | 0.754 | 0.999 | 0.856 | 0.997 | 0.258 | 0.926 | 0.935 | 0.966 | 0.440 | 0.973 | 0.073 | 0.901 |
| Malic acid (peak 2) | 0.778 | 0.999 | 0.746 | 0.997 | 0.301 | 0.926 | 0.942 | 0.966 | 0.350 | 0.973 | 0.121 | 0.901 |
| Malonic acid | 0.924 | 0.999 | 0.829 | 0.997 | 0.642 | 0.986 | 0.831 | 0.966 | 0.973 | 0.973 | 0.210 | 0.901 |
| Methionine | 0.586 | 0.999 | 0.469 | 0.997 | 0.162 | 0.821 | 0.305 | 0.966 | 0.225 | 0.973 | 0.884 | 0.955 |
| Methylthioacetic acid | 0.080 | 0.999 | 0.427 | 0.997 | 0.103 | 0.821 | 0.282 | 0.966 | 0.909 | 0.973 | 0.046 | 0.901 |
| Myristic acid | 0.630 | 0.999 | 0.558 | 0.997 | 0.687 | 0.986 | 0.392 | 0.966 | 0.470 | 0.973 | 0.155 | 0.901 |
| Myristoleic acid | 0.830 | 0.999 | 0.736 | 0.997 | 0.866 | 0.986 | 0.611 | 0.966 | 0.310 | 0.973 | 0.529 | 0.901 |
| Nicotinamide | 0.471 | 0.999 | 0.633 | 0.997 | 0.934 | 0.986 | 0.244 | 0.966 | 0.003 | 0.268 | 0.838 | 0.951 |
| Nicotinamide adenine dinucleotide phosphate | 0.370 | 0.999 | 0.665 | 0.997 | 0.177 | 0.828 | 0.544 | 0.966 | 0.878 | 0.973 | 0.498 | 0.901 |
| Nicotinic acid | 0.198 | 0.999 | 0.313 | 0.997 | 0.106 | 0.821 | 0.779 | 0.966 | 0.775 | 0.973 | 0.861 | 0.955 |
| Norvaline | 0.384 | 0.999 | 0.672 | 0.997 | 0.654 | 0.986 | 0.804 | 0.966 | 0.302 | 0.973 | 0.256 | 0.901 |
| Octanoic acid | 0.966 | 0.999 | 0.740 | 0.997 | 0.386 | 0.986 | 0.226 | 0.966 | 0.785 | 0.973 | 0.579 | 0.901 |
| Ornithine | 0.065 | 0.999 | 0.480 | 0.997 | 0.064 | 0.821 | 0.809 | 0.966 | 0.382 | 0.973 | 0.077 | 0.901 |
| Oxalic acid | 0.615 | 0.999 | 0.306 | 0.997 | 0.043 | 0.821 | 0.305 | 0.966 | 0.642 | 0.973 | 0.700 | 0.901 |
| para-toluic acid | 0.744 | 0.999 | 0.587 | 0.997 | 0.552 | 0.986 | 0.655 | 0.966 | 0.119 | 0.765 | 0.903 | 0.955 |
| Pentadecanoic acid | 0.458 | 0.999 | 0.645 | 0.997 | 0.108 | 0.821 | 0.590 | 0.966 | 0.808 | 0.973 | 0.666 | 0.901 |
| Phenethyl acetate | 0.129 | 0.999 | 0.885 | 0.997 | 0.035 | 0.821 | 0.591 | 0.966 | 0.844 | 0.973 | 0.337 | 0.901 |
| Phenylalanine | 0.537 | 0.999 | 0.417 | 0.997 | 0.822 | 0.986 | 0.040 | 0.966 | 0.598 | 0.973 | 0.027 | 0.901 |
| Proline | 0.953 | 0.999 | 0.786 | 0.997 | 0.818 | 0.986 | 0.733 | 0.966 | 0.636 | 0.973 | 0.510 | 0.901 |
| Pyroglutamic acid | 0.649 | 0.999 | 0.218 | 0.997 | 0.542 | 0.986 | 0.731 | 0.966 | 0.030 | 0.423 | 0.535 | 0.901 |
| Pyruvic acid | 0.405 | 0.999 | 0.867 | 0.997 | 0.295 | 0.926 | 0.231 | 0.966 | 0.488 | 0.973 | 0.481 | 0.901 |
| Salicylic acid | 0.025 | 0.999 | 0.243 | 0.997 | 0.134 | 0.821 | 0.101 | 0.966 | 0.024 | 0.423 | 0.348 | 0.901 |
| Serine | 0.835 | 0.999 | 0.981 | 0.997 | 0.940 | 0.986 | 0.413 | 0.966 | 0.538 | 0.973 | 0.474 | 0.901 |
| Stearic acid | 0.695 | 0.999 | 0.482 | 0.997 | 0.811 | 0.986 | 0.496 | 0.966 | 0.449 | 0.973 | 0.305 | 0.901 |
| Suberic acid | 0.453 | 0.999 | 0.828 | 0.997 | 0.293 | 0.926 | 0.721 | 0.966 | 0.949 | 0.973 | 0.898 | 0.955 |
| Succinic acid | 0.967 | 0.999 | 0.537 | 0.997 | 0.946 | 0.986 | 0.647 | 0.966 | 0.909 | 0.973 | 0.340 | 0.901 |
| Tartaric acid | 0.947 | 0.999 | 0.516 | 0.997 | 0.609 | 0.986 | 0.981 | 0.989 | 0.520 | 0.973 | 0.779 | 0.921 |
| Tetracosanoic acid | 0.888 | 0.999 | 0.196 | 0.997 | 0.423 | 0.986 | 0.905 | 0.966 | 0.445 | 0.973 | 0.668 | 0.901 |
| Threonine | 0.807 | 0.999 | 0.633 | 0.997 | 0.590 | 0.986 | 0.933 | 0.966 | 0.029 | 0.423 | 0.256 | 0.901 |
| trans-10-heptadecenoic acid | 0.769 | 0.999 | 0.708 | 0.997 | 0.851 | 0.986 | 0.942 | 0.966 | 0.645 | 0.973 | 0.450 | 0.901 |
| trans-10-nonadecenoic acid | 0.830 | 0.999 | 0.377 | 0.997 | 0.864 | 0.986 | 0.469 | 0.966 | 0.823 | 0.973 | 0.790 | 0.921 |
| trans-11-eicosenoic acid | 0.768 | 0.999 | 0.342 | 0.997 | 0.876 | 0.986 | 0.593 | 0.966 | 0.842 | 0.973 | 0.869 | 0.955 |
| trans-7-nonadecenoic acid | 0.434 | 0.999 | 0.138 | 0.997 | 0.891 | 0.986 | 0.859 | 0.966 | 0.415 | 0.973 | 0.644 | 0.901 |
| trans-9-octadecenoic acid | 0.971 | 0.999 | 0.356 | 0.997 | 0.909 | 0.986 | 0.564 | 0.966 | 0.790 | 0.973 | 0.567 | 0.901 |
| trans-cinnamic acid | 0.857 | 0.999 | 0.221 | 0.997 | 0.855 | 0.986 | 0.853 | 0.966 | 0.958 | 0.973 | 0.310 | 0.901 |
| Tricosanoic acid | 0.492 | 0.999 | 0.724 | 0.997 | 0.286 | 0.926 | 0.863 | 0.966 | 0.355 | 0.973 | 0.545 | 0.901 |
| Tridecane | 0.181 | 0.999 | 0.062 | 0.997 | 0.278 | 0.926 | 0.219 | 0.966 | 0.057 | 0.516 | 0.350 | 0.901 |
| Tryptophan | 0.923 | 0.999 | 0.470 | 0.997 | 0.564 | 0.986 | 0.803 | 0.966 | 0.120 | 0.765 | 0.052 | 0.901 |
| Tyrosine | 0.857 | 0.999 | 0.870 | 0.997 | 0.272 | 0.926 | 0.077 | 0.966 | 0.528 | 0.973 | 0.386 | 0.901 |
| Undecanoic acid | 0.889 | 0.999 | 0.444 | 0.997 | 0.489 | 0.986 | 0.817 | 0.966 | 0.572 | 0.973 | 0.442 | 0.901 |
| Valine | 0.894 | 0.999 | 0.973 | 0.997 | 0.866 | 0.986 | 0.795 | 0.966 | 0.664 | 0.973 | 0.256 | 0.901 |
| Vitamin E | 0.162 | 0.999 | 0.644 | 0.997 | 0.005 | 0.650 | 0.796 | 0.966 | 0.369 | 0.973 | 0.883 | 0.955 |
| Models adjusted for age, sex, deprivation index, Apolipoprotein E -ε4, body fat percentage (standardised by sex), education, physical activity, metabolic syndrome, polypharmacy, smoking status, alcohol intake. | | | | | | | | | | | | |
